# GLS1 governs vascular smooth muscle cell phenotypic switching and aortic dissection via glutamate metabolism

**DOI:** 10.1172/jci.insight.203575

**Published:** 2026-04-23

**Authors:** Wei Xie, Chen Ning, Chen Lu, Dongjin Wang, Shuang Zhao, Tianyu Song, Hailong Cao

**Affiliations:** 1 Department of Cardiac Surgery, Zhongda Hospital, School of Medicine, Southeast University, Nanjing, Jiangsu, China.; 2 School of Pharmacy, Nanjing Medical University, Nanjing, Jiangsu, China.; 3 Department of Cardiac Surgery, Nanjing Drum Tower Hospital, Affiliated Hospital of Medical School, Nanjing University, Nanjing, Jiangsu, China.; 4 School of Medicine, Nanjing University of Chinese Medicine, Nanjing, Jiangsu, China.

**Keywords:** Cardiology, Vascular biology, Molecular pathology, Muscle, Therapeutics

## Abstract

Aortic dissection (AD) is a catastrophic vascular emergency with high mortality, and current pharmacological interventions to prevent its progression are limited. Vascular smooth muscle cells (VSMCs) undergo a pathological phenotypic switch from a contractile to a synthetic state during AD, compromising aortic wall integrity; however, the underlying metabolic mechanisms remain poorly understood. In this study, we performed integrative transcriptomic analyses and identified glutaminase 1 (GLS1) as a key regulator of VSMC phenotypic switching in AD. GLS1 expression was significantly downregulated in VSMCs from both human AD aortic tissues and mouse models. Functionally, GLS1 deficiency promoted PDGF-BB–induced VSMC dedifferentiation in vitro. Smooth muscle cell–specific *Gls1*-knockout (*Gls1^SMKO^*) mice exhibited aggravated AD after β-aminopropionitrile treatment, whereas VSMC-specific GLS1 overexpression improved the contractile phenotype and reduced AD incidence. Mechanistically, GLS1 downregulation impaired glutamate metabolism, leading to reduced levels of glutathione and α-ketoglutarate. This metabolic disruption promoted reactive oxygen species accumulation and mitochondrial dysfunction, ultimately triggering VSMC phenotypic switching. Furthermore, we found that *GLS1* transcription was repressed by retinoic acid receptor-α (RARα). Pharmacological inhibition of RARα with AR7 restored GLS1 expression, ameliorated VSMC phenotypic switching, and conferred protection against AD. These findings reveal a critical role of GLS1-mediated glutamate metabolism in VSMC phenotypic switching and suggest a promising therapeutic strategy for AD.

## Abstract 2

<strong>A key metabolic enzyme, GLS1, helps protect the aorta by keeping muscle cells healthy, offering a promising new treatment strategy for aortic dissection.</strong>

## Graphical abstract

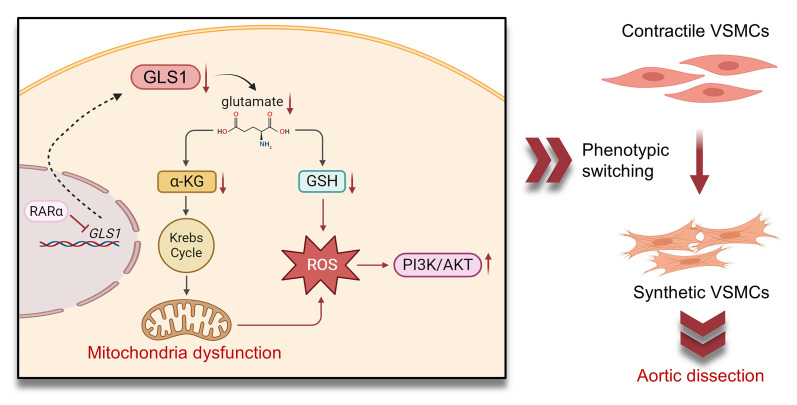

## Introduction

Aortic dissection (AD) is a catastrophic aortic emergency characterized by an intimal tear through which blood enters the medial layer, creating a true and a false lumen. The architectural disruption of the aortic wall can acutely compromise perfusion to vital organs, frequently leading to fatal outcomes (1). Epidemiological data indicate that, in untreated acute ascending dissections, mortality rises by 1%–2% per hour following symptom onset, underscoring the critical importance of immediate surgical intervention (2). Nevertheless, effective pharmacological interventions to prevent or delay AD progression are currently lacking, underscoring the critical need to elucidate the molecular mechanisms underlying the disease and identify novel therapeutic targets.

Vascular smooth muscle cells (VSMCs), the predominant cellular component of the aortic media, play a fundamental role in maintaining vessel wall integrity and function (3). VSMCs demonstrate remarkable phenotypic plasticity, transitioning from contractile to synthetic state in response to microenvironmental stimuli. During AD development and progression, VSMCs undergo a phenotypic shift toward the synthetic state, characterized by downregulation of contractile proteins and related genes and heightened matrix metalloproteinase (MMP) activity (4). These alterations collectively compromise extracellular matrix (ECM) architecture, accelerate elastic fiber degradation, and ultimately weaken aortic wall stability (5). Although maintaining contractile phenotype of VSMCs is recognized as a promising therapeutic approach for AD, the underlying mechanisms governing VSMC phenotypic switching and corresponding intervention approaches remain to be fully elucidated and developed.

Emerging evidence has revealed that mitochondrial dysfunction–associated metabolic alterations play pivotal regulatory roles in VSMC phenotypic switching: several metabolic enzymes, including aconitase-2 (6), aldehyde dehydrogenase 2 (7), oxoglutarate dehydrogenase (8), and pyruvate kinase M (9), have been demonstrated to critically participate in the pathogenesis of aortic aneurysms and dissections. However, the precise mechanisms underlying the role of mitochondrial homeostasis disruption in VSMC phenotypic switching remain to be elucidated. Glutaminase 1 (GLS1), a mitochondrial enzyme responsible for converting glutamine to glutamate, serves as a key regulator of glutamine metabolism. While GLS1 upregulation has been well documented in various malignancies, where it supports anabolic growth and bioenergetic requirements (10, 11), its role in cardiovascular disease appears to be context dependent. Emerging evidence suggests that GLS1 may promote plaque stabilization in atherosclerosis (12), whereas fibroblast-specific GLS1 deletion attenuates myocardial fibrosis in pressure-overload heart failure (13). However, the potential involvement of GLS1 in AD pathogenesis remains unexplored.

In this study, we identify GLS1 as a previously unrecognized regulator of VSMC phenotypic switching during AD progression. The expression of GLS1 in VSMCs is markedly reduced in both human AD specimens and rodent models. Loss of GLS1 perturbs glutamine metabolism to induce mitochondrial dysfunction and oxidative stress, thereby activating the PI3K/AKT pathway to drive VSMC phenotypic switching. Notably, we demonstrated that retinoic acid receptor-α (RARα) functions as a transcriptional repressor of GLS1. Pharmacological inhibition of RARα with AR7 restores GLS1 expression and significantly improves AD pathology, highlighting a potential therapeutic strategy for AD.

## Results

### Decreased GLS1 in VSMCs is associated with AD.

**Figure 1:**
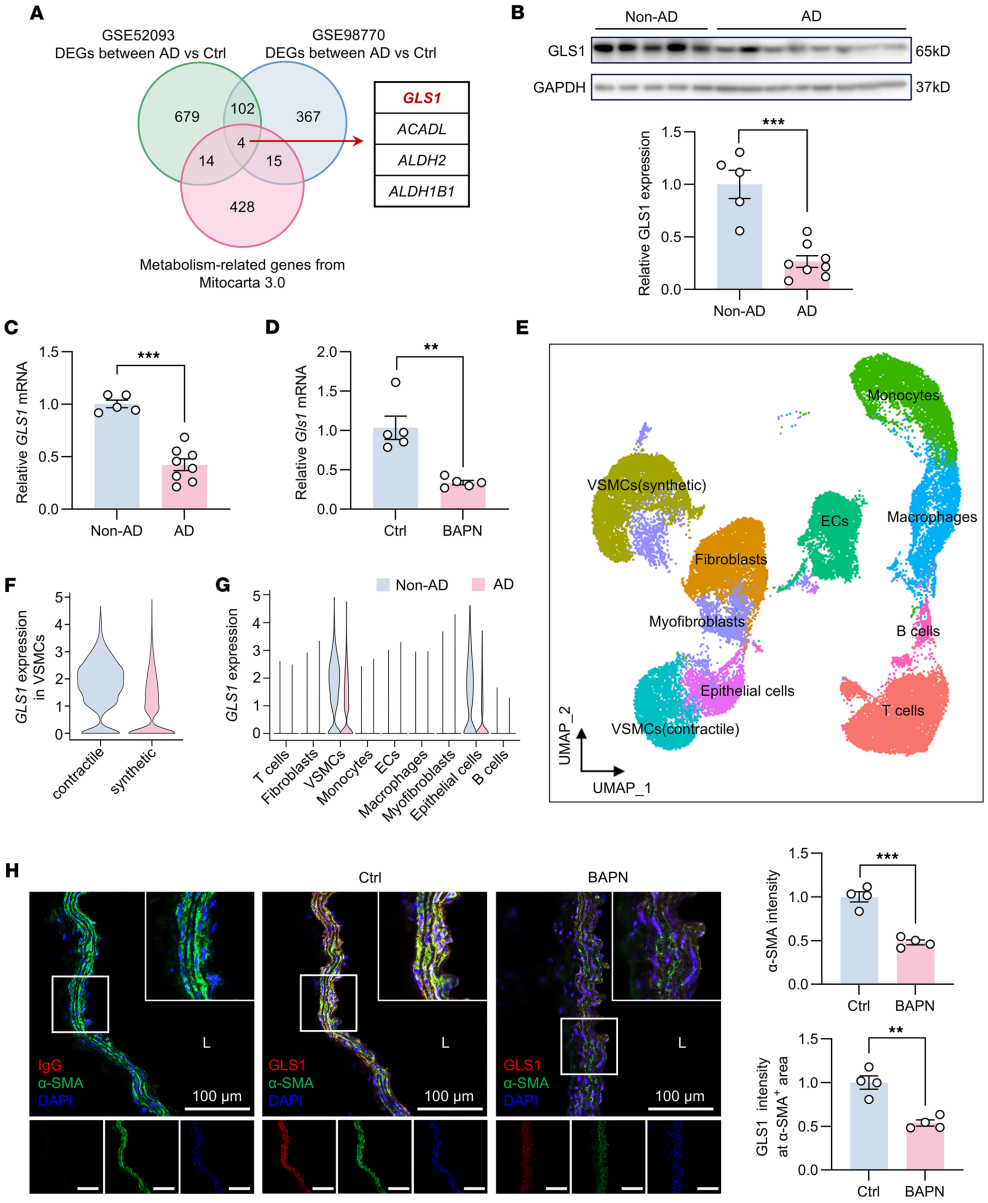
Expression of GLS1 in VSMCs is decreased in AD. (**A**) Venn diagram showing the 4 overlap genes (*GLS1*, glutaminase 1; *ACADL*, long-chain acylase A dehydrogenase; *ALDH2*, aldehyde dehydrogenase 2; *ALDH1B1*, aldehyde dehydrogenase 1 family member B1) between differentially expressed genes (DEGs) in human AD database (GSE52093, GSE98770) and metabolic-related genes from Mitocarta3. (**B**) Western blot analysis of GLS1 expression in aorta from non-AD groups and AD patients. (**C**) mRNA expression of *GLS1* in aorta from non-AD groups and AD patients was detected by real-time qPCR. (**D**) Three-week-old male C57BL/6 mice were treated with β-aminopropionitrile (BAPN) in drinking water (1 g/kg/d) for 4 weeks. mRNA expression of *Gls1* in mouse aorta was detected by real-time qPCR. (**E**) Uniform manifold approximation and projection (UMAP) visualization of single cells from human aortic tissues (GSE213740). Cells were partitioned into 10 major lineages: T cell, fibroblast, VSMC (contractile), VSMC (synthetic), monocyte, endothelial cell, macrophage, myofibroblast, epithelial cell, and B cell. (**F**) *GLS1* expression among distinct cellular populations between non-AD groups and AD patients. (**G**) *GLS1* expression among distinct cell types between non-AD groups and AD patients. (**H**) Three-week-old male C57BL/6 mice were treated with BAPN in drinking water (1 g/kg/d) for 4 weeks. Immunofluorescence staining for GLS1 (red), α-SMA (green), and DAPI (blue) in mouse aorta. Scale bars: 100 μm. Data are presented as mean ± SEM. Statistical analysis was performed using unpaired, 2-tailed Student’s *t* test (**B**–**D**, **G**, and **H**). **P < 0.01, ***P < 0.001.

To identify candidate genes potentially involved in VSMC phenotypic switching in AD, we integrated 2 transcriptomic datasets from AD patients (Gene Expression Omnibus [GEO] GSE52093 and GSE98770) and cross-referenced them with mitochondrial metabolism–related gene sets (MitoCarta3.0, ref. 14). As shown by Venn diagram, *GLS1*, *ACADL*, *ALDH2*, and *ALDH1B1* were consistently shared across the datasets, identifying them as key candidate genes linking mitochondria-related metabolism and VSMC phenotypic switching to AD (Figure 1A). Notably, *ACADL* and *ALDH2* have been previously associated with aortic aneurysm/dissection (7, 15), and *ALDH1B1* shares high sequence/structure conservation with *ALDH2* (16). Given that the precise role of GLS1 in AD remains undefined, we here sought to investigate its impact on VSMC phenotypic switching and AD pathogenesis.

Transcriptomic analysis of AD patient datasets (GSE52093 and GSE98770) revealed reduced *GLS1* expression in AD tissues compared with controls (Supplemental Figure 1, A–D; supplemental material available online with this article; https://doi.org/10.1172/jci.insight.203575DS1). Importantly, we verified that the aortic GLS1 protein levels were significantly decreased in AD patients (Figure 1B). Meanwhile, the mRNA levels of *GLS1* were reduced in aortic tissues from both AD patients and β-aminopropionitrile–induced (BAPN-induced) AD mice (Figure 1, C and D). To determine GLS1 expression patterns within the vascular wall, we analyzed a single-cell RNA sequencing dataset of AD patients (GSE213740). Uniform manifold approximation and projection (UMAP) visualization identified 10 major cell lineages (Figure 1E). The 2 separate populations of VSMCs were identified as contractile and synthetic phenotype, respectively, according the VSMC phenotypic marker genes (Supplemental Figure 1, E–G). Notably, we observed that expression of *GLS1* was obviously decreased in the synthetic VSMCs (Figure 1F). Also, it was highly expressed in VSMCs from non-AD subjects and exhibited a striking reduction in AD patients (Figure 1G). Immunofluorescence also demonstrated decreased GLS1 in the tunica media of aortic tissues from mouse AD models (Figure 1H). These results suggested that the downregulation of GLS1 in VSMCs may participate in AD development.

### GLS1 regulates VSMC phenotypic switching in vitro.

**Figure 2:**
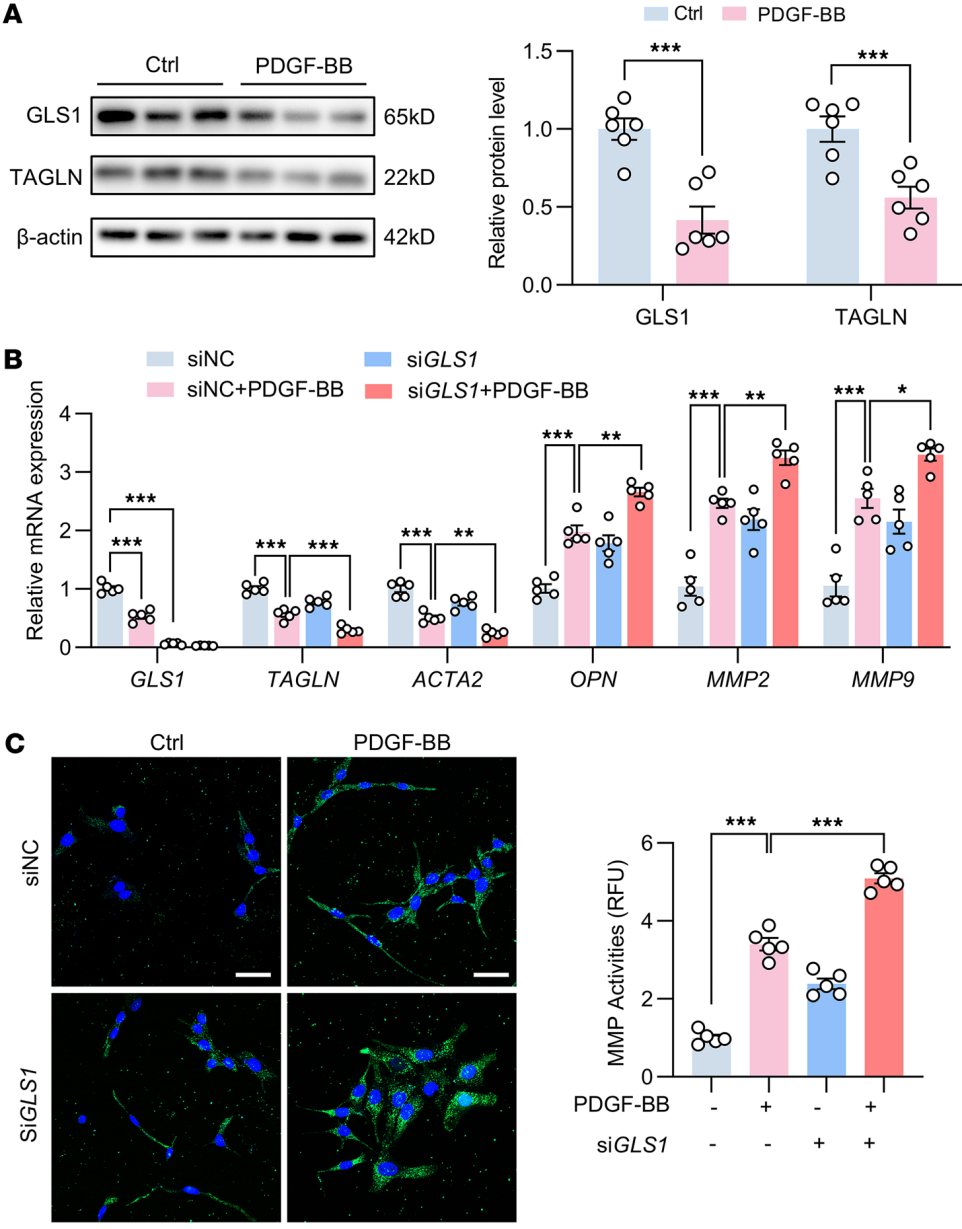
GLS1 knockdown promotes phenotypic switching of VSMCs in response to PDGF-BB. (**A**) Human aortic smooth muscle cells (HASMCs) were treated with PDGF-BB. Western blot analysis of GLS1 and TAGLN expression. (**B** and **C**) HASMCs were transfected with siRNA against *GLS1* (si*GLS1*) or negative control (siNC), and then treated with PDGF-BB. (**B**) The mRNA expression of *GLS1*, *TAGLN*, *ACTA2*, *OPN*, *MMP2*, and *MMP9* in HASMCs was detected by real-time qPCR. (**C**) Immunofluorescence images of in situ zymography (DQ gelatin) in HASMCs. MMP activity (green) was quantified by immunofluorescence intensity. Scale bars: 50 μm. (Blue, DAPI.) Data are presented as mean ± SEM. Statistical analysis was performed using unpaired, 2-tailed Student’s *t* test (**A**), or 1-way ANOVA followed by Tukey’s multiple-comparison test (**B** and **C**). *P < 0.05, **P < 0.01, ***P < 0.001.

**Figure 3:**
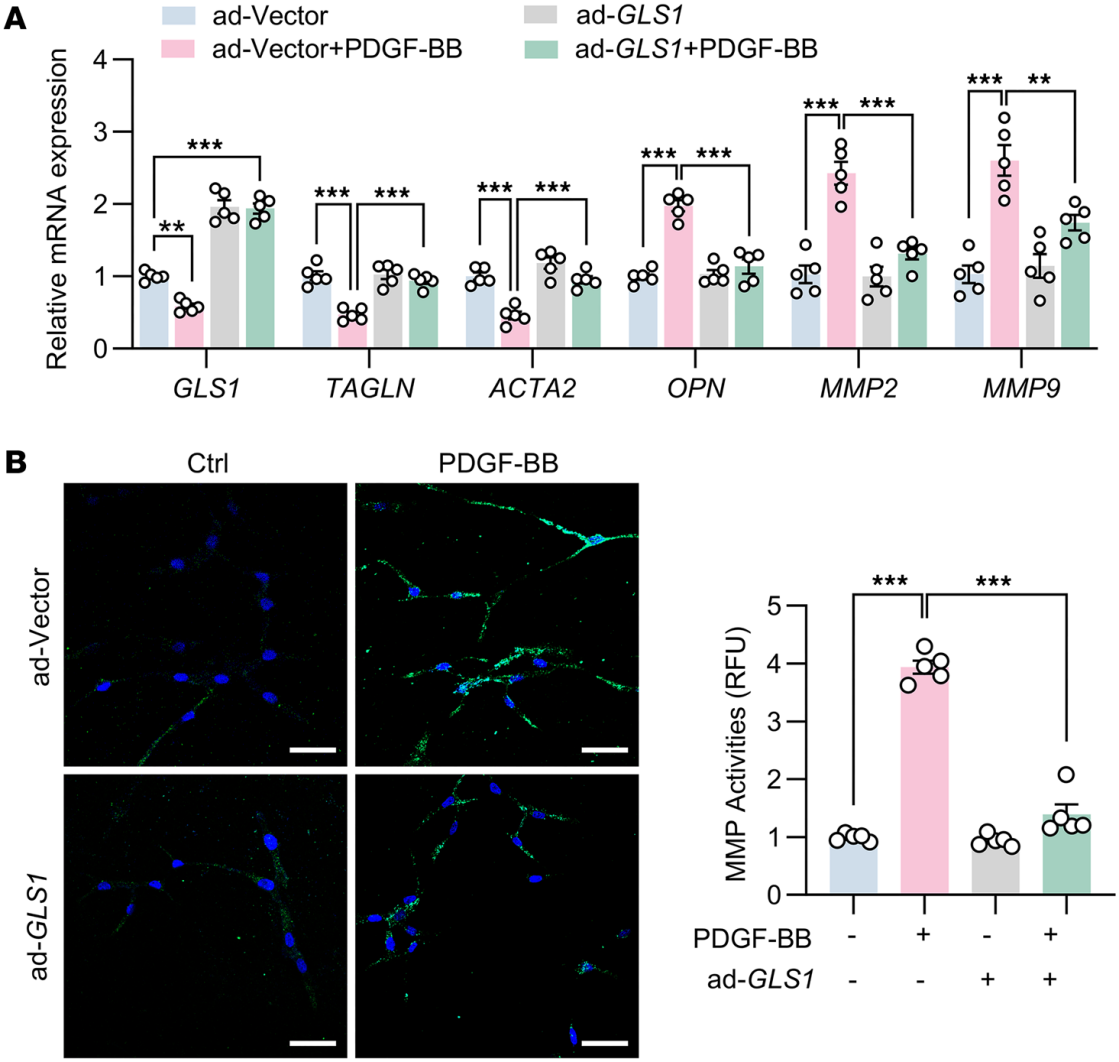
GLS1 overexpression preserves the contractile phenotype of VSMCs in response to PDGF-BB. (**A** and **B**) HASMCs were infected with adenovirus containing empty vector (ad-Vector) or GLS1-encoding plasmids (ad-*GLS1*), and then treated with PDGF-BB. (**A**) The mRNA expression of *GLS1*, *TAGLN*, *ACTA2*, *OPN*, *MMP2*, and *MMP9* in HASMCs was detected by real-time qPCR. (**B**) Immunofluorescence images of in situ zymography (DQ gelatin) in HASMCs. MMP activity (green) was quantified by immunofluorescence intensity. Scale bars: 50 μm. (Blue: DAPI.) Data are presented as mean ± SEM. Statistical analysis was performed using 1-way ANOVA followed by Tukey’s multiple-comparison test (**A** and **B**). **P < 0.01, ***P < 0.001.

In physiological conditions, VSMCs maintain a differentiated contractile phenotype. However, they dedifferentiate and convert to a synthetic phenotype in response to pathological stimuli, a process recognized as a pivotal mechanism in AD pathogenesis (17). Next, we used PDGF-BB to stimulate human aortic smooth muscle cells (HASMCs) to induce VSMC dedifferentiation and found that GLS1 was decreased upon PDGF-BB stimulation (Figure 2A). To determine whether GLS1 participated in VSMC dedifferentiation, we used siRNA to knock down *GLS1* in HASMCs (Supplemental Figure 2A), followed by PDGF-BB treatment. The contractile-related genes (*TAGLN* and *ACTA2*) and synthetic-related gene (*OPN*) in HASMCs were detected, and the results showed that *GLS1* knockdown further inhibited the mRNA levels of *TAGLN* and *ACTA2*, and promoted *OPN* mRNA levels upon PDGF-BB stimulation (Figure 2B). Meanwhile, Western blot analysis revealed that *GLS1* knockdown further inhibited TAGLN expression and enhanced OPN expression in PDGF-BB–treated HASMCs (Supplemental Figure 2B). Also, in situ zymography revealed that knockdown of *GLS1* promoted the MMP activities induced by PDGF-BB (Figure 2C). To further evaluate the role of GLS1 during VSMC phenotypic switching, we used adenovirus to overexpress GLS1 in HASMCs (Supplemental Figure 3A). We found that the VSMC phenotypic switching induced by PDGF-BB could be improved by GLS1 overexpressed in HASMCs, as evidenced by increased *TAGLN* and *ACTA2*, and decreased *OPN* mRNA levels upon GLS1 overexpression (Figure 3A), and similar results were obtained by Western blot analysis (Supplemental Figure 3B). GLS1 overexpression also inhibited MMP activities in the condition of PDGF-BB treatment (Figure 3B). Collectively, these findings demonstrated that downregulation of GLS1 promotes VSMC dedifferentiation into a synthetic phenotype, and GLS1 overexpression attenuates pathological VSMC phenotypic switching in vitro.

### VSMC-specific GLS1 overexpression ameliorates BAPN-induced AD.

**Figure 4:**
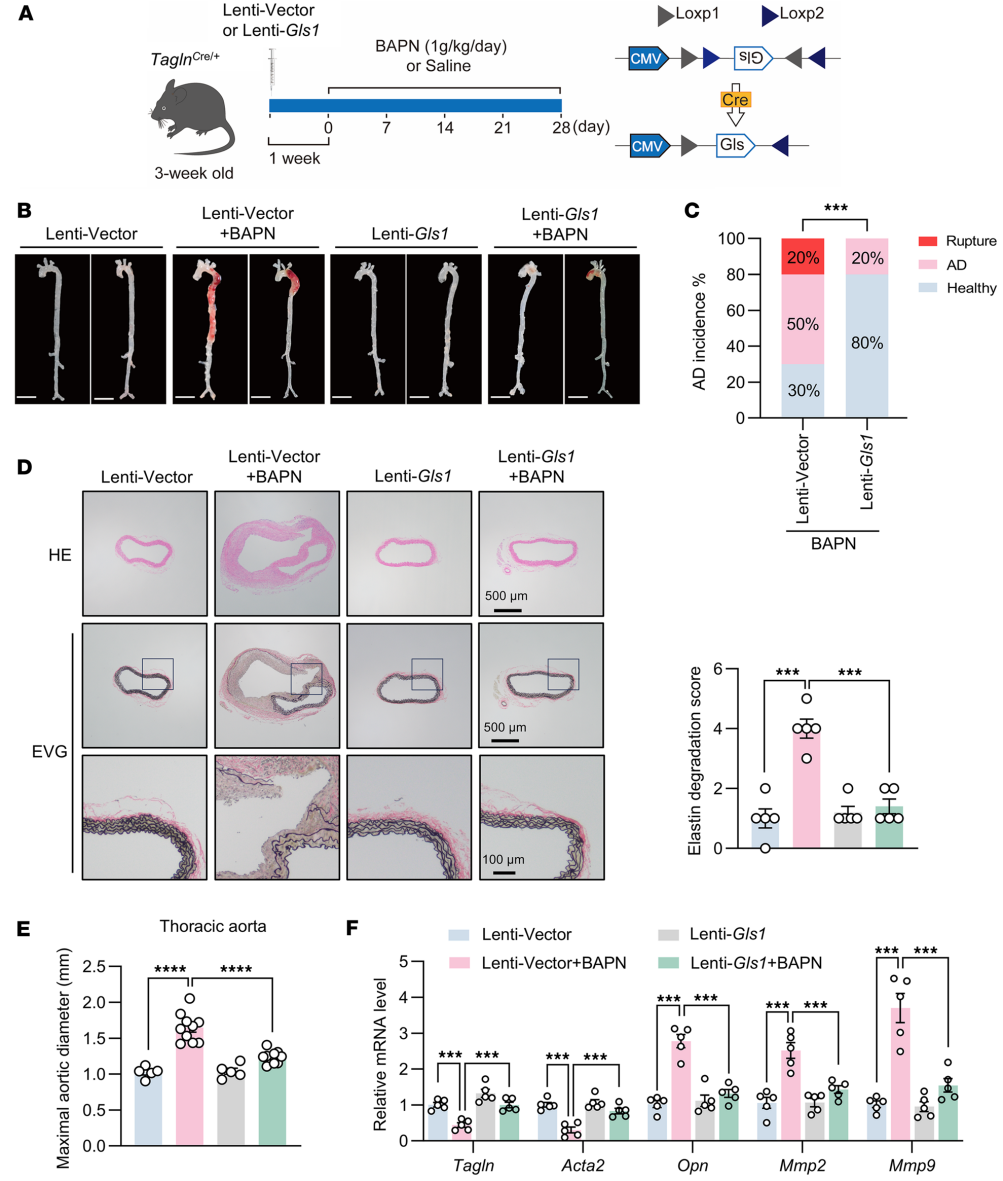
Overexpression of GLS1 in VSMCs attenuates AD in BAPN-treated mice. (**A**) Three-week-old male *Tagln^Cre/+^* mice were intravenously injected with lentivirus containing control vector or reverse *Gls1* sequence with 2 *loxP* sites. After 1 week, BAPN (1 g/kg/d) was given in drinking water for 4 weeks. (**B**) Representative morphology of aortae from mice in the control group (*n* = 5) and the BAPN-treated group (*n* = 10). Scale bars: 5 mm. (**C**) The incidence of aortic dissection and rupture in BAPN-treated mice (*n* = 10). (**D**) Hematoxylin and eosin (H&E) and elastic Verhoeff–van Gieson (EVG) staining of aorta from control or BAPN-treated mice, and quantifications of elastic fiber breaks. Scale bars: 500 μm (top and middle panels), 100 μm (bottom panels). (**E**) Quantification of maximal diameters measured ex vivo of aorta from control and BAPN-treated mice. (**F**) The mRNA expression of *Tagln*, *Acta2*, *Opn*, *Mmp2*, and *Mmp9* in aorta from control and BAPN-treated mice was detected by real-time qPCR. Data are presented as mean ± SEM. Statistical analysis was performed using Fisher’s exact test (**C**), or 1-way ANOVA followed by Tukey’s multiple-comparison test (**E** and **F**). ***P < 0.001, ****P < 0.0001.

To assess the role of GLS1 in VSMCs during AD, we constructed lentivirus carrying empty vector (Lenti-Vector) or *Gls1* overexpression plasmid (Lenti-*Gls1*) containing 2 *loxP* sites that can be recognized by Cre recombinase. Then, 3-week-old male *Tagln^Cre/+^* mice received intravenous injections of either Lenti-Vector or Lenti-*Gls1*. One week after injection, AD was induced by administration of BAPN in drinking water for 4 weeks (Figure 4A). The expression of exogenous GLS1 in aortic VSMCs was verified by immunofluorescence staining with FLAG (Supplemental Figure 4A). Notably, we found that delivery of Lenti-*Gls1* could attenuate AD lesion induced by BAPN (Figure 4B), and significantly decreased the incidence of aortic dissection and rupture (Figure 4C). Hematoxylin and eosin (H&E) staining showed reduced aortic dilatation in Lenti-*Gls1* mice fed by BAPN, while elastic Verhoeff–van Gieson (EVG) staining also demonstrated that aortic media degeneration was improved by delivery of Lenti-*Gls1* (Figure 4D). Meanwhile, overexpression of GLS1 significantly mitigated the dilatation of thoracic aorta diameter induced by BAPN (Figure 4E). The mRNA levels of VSMC phenotypic switching–related genes showed that delivery of Lenti-*Gls1* could reverse the reduced expression of contractile-related genes (*Tagln* and *Acta2*) and enhanced expression of synthetic-related gene (*Opn*, *Mmp2*, and *Mmp9*) in aorta from BAPN-fed mice (Figure 4F). These data suggested that GLS1 overexpression could improve VSMC phenotypic switching and protect against AD in vivo.

### GLS1 deficiency in VSMCs aggravates AD in BAPN-treated mice.

**Figure 5:**
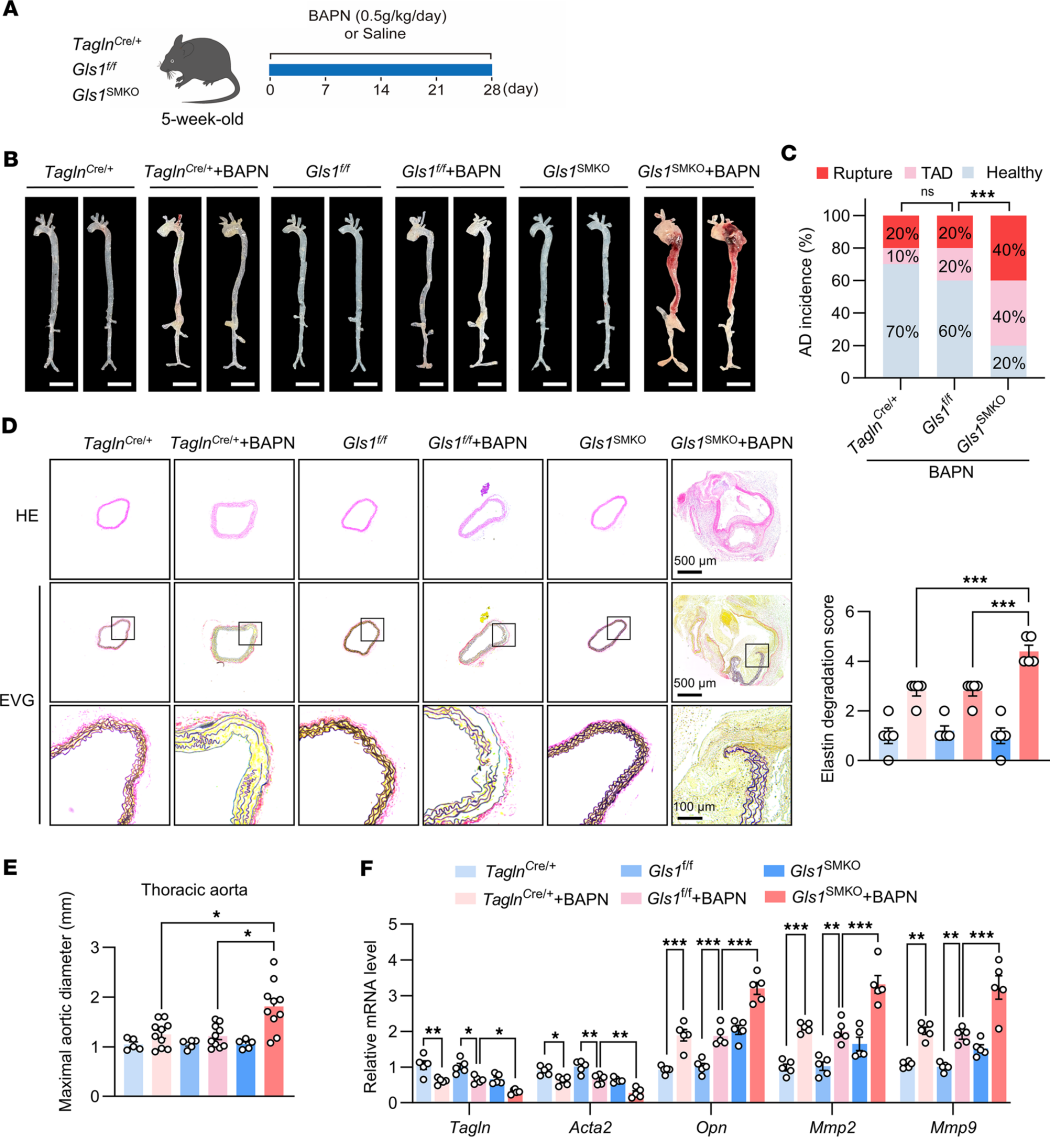
GLS1 deficiency in VSMCs aggravates AD in BAPN-treated mice. (**A**) Five-week-old male *Tagln^Cre/+^*, *Gls1^fl/fl^* , and *Gls1^SMKO^* mice were given BAPN (0.5 g/kg/d) in drinking water for 4 weeks. (**B**) Representative morphology of aortae from *Tagln^Cre/+^*, *Gls1^fl/fl^*, and *Gls1^SMKO^* mice under control (*n* = 5) conditions or after BAPN treatment (*n* = 10). Scale bars: 5 mm. (**C**) The incidence of aortic dissection and rupture in BAPN-treated mice (*n* = 10). (**D**) H&E and EVG staining of aorta from *Tagln^Cre/+^*, *Gls1^fl/fl^*, and *Gls1^SMKO^* mice under control conditions or after BAPN-treatment, and quantifications of elastic fiber breaks. Scale bars: 500 μm (top and middle panels), 100 μm (bottom panels). (**E**) Quantification of maximal aortic diameters measured ex vivo in *Tagln^Cre/+^*, *Gls1^fl/fl^*, and *Gls1^SMKO^* mice under control conditions or after BAPN treatment. (**F**) The mRNA expression of *Tagln*, *Acta2*, *Opn*, *Mmp2*, and *Mmp9* in aortic tissues from *Tagln^Cre/+^*, *Gls1^fl/fl^*, and *Gls1^SMKO^* mice under control conditions or after BAPN treatment was detected by real-time qPCR. Data are presented as mean ± SEM. Statistical analysis was performed using Fisher’s exact test (**C**), or 1-way ANOVA followed by Tukey’s multiple-comparison test (**E** and **F**). *P < 0.05, **P < 0.01, ***P < 0.001.

To assess the effect of GLS1 deficiency in VSMCs during AD, we used smooth muscle cell–specific *Gls1*-knockout mice (*Gls1^SMKO^*) and their littermate controls (*Tagln^Cre/+^* and *Gls1^fl/fl^*). Five-week-old male *Tagln^Cre/+^*, *Gls1^fl/fl^*, and *Gls1^SMKO^* mice were given BAPN in drinking water for 4 weeks to induce AD (Figure 5A). Notably, GLS1 deficiency markedly exacerbated BAPN-induced AD lesions (Figure 5B) and significantly increased the incidence of aortic dissection and rupture (Figure 5C). H&E staining revealed more severe aortic dilatation in *Gls1^SMKO^* mice exposed to BAPN, while EVG staining demonstrated pronounced aortic media degeneration in comparison with controls (Figure 5D). Moreover, GLS1 deficiency significantly increased thoracic aortic diameters after BAPN treatment (Figure 5E). Analysis of VSMC phenotypic switching–related genes showed that GLS1 deficiency further suppressed the expression of contractile markers (*Tagln* and *Acta2*) and enhanced the expression of synthetic markers (*Opn*, *Mmp2*, and *Mmp9*) in aortae from BAPN-fed mice (Figure 5F). These data indicate that GLS1 deficiency promotes VSMC phenotypic switching and aggravates AD in vivo.

### GLS1-mediated glutamate metabolism regulates PI3K/AKT signaling to modulate VSMC phenotypic switching.

**Figure 6:**
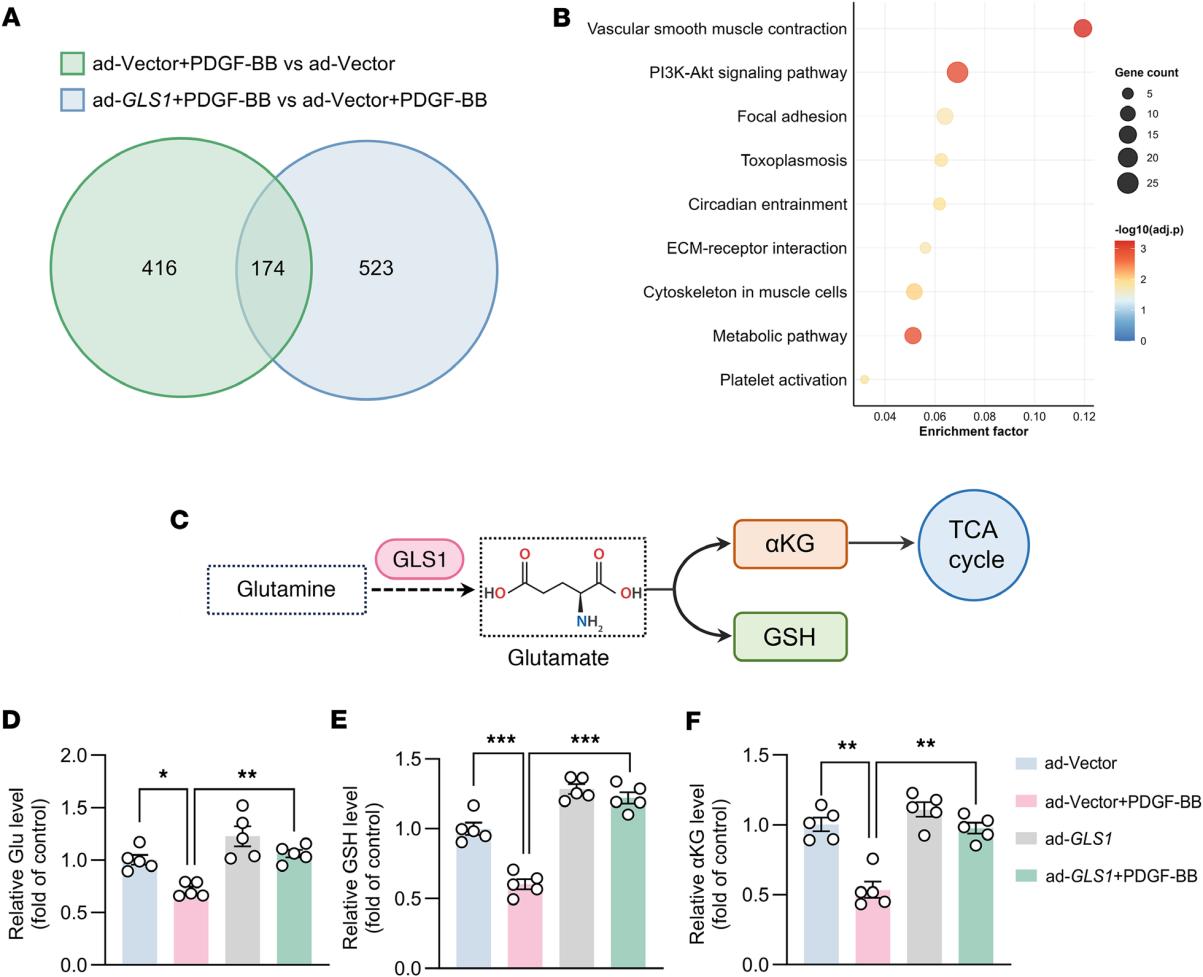
GLS1 regulates glutamine metabolism during VSMC phenotypic switching. (**A**) RNA sequencing was performed on HASMCs infected with adenovirus containing empty vector (ad-Vector) or GLS1-encoding plasmids (ad-*GLS1*) and then treated with PDGF-BB. Venn diagram showing the overlap DEGs between ad-Vector+PDGF-BB versus ad-Vector and ad-*GLS1*+PDGF-BB versus ad-Vector+PDGF-BB (*n* = 3). (**B**) KEGG enrichment analysis of these overlap DEGs. (**C**) Schematic diagram of GLS1-related metabolic pathway for glutamine. (**D**–**F**) HASMCs were infected with adenovirus containing empty vector (ad-Vector) or GLS1-encoding plasmids (ad-*GLS1*), and then treated with PDGF-BB. (**D**) The content of glutamate in HASMCs. (**E**) The content of glutathione (GSH) in HASMCs. (**F**) The content of α-ketoglutarate (αKG) in HASMCs. Data are presented as mean ± SEM. Statistical analysis was performed using 1-way ANOVA followed by Tukey’s multiple-comparison test (**D**–**F**). *P < 0.05, **P < 0.01, ***P < 0.001.

To further investigate the underlying regulatory mechanism for GLS1-related VSMC phenotypic switching, we performed RNA sequencing in HASMCs with GLS1 overexpression by adenovirus with or without PDGF-BB treatment to scan the potential genes regulated by GLS1. As shown by Venn diagram, 174 genes appeared to be differently expressed both in ad-Vector+PDGF-BB versus ad-Vector and in ad-*GLS1*+PDGF-BB versus ad-Vector+PDGF-BB (Figure 6A). Kyoto Encyclopedia of Genes and Genomes (KEGG) enrichment analysis revealed the 174 differentially expressed genes (DEGs) mainly involved in vascular smooth muscle contraction and PI3K/AKT signaling (Figure 6B), indicating that GLS1 may regulate VSMC phenotypic switching via the PI3K/AKT pathway.

**Figure 7:**
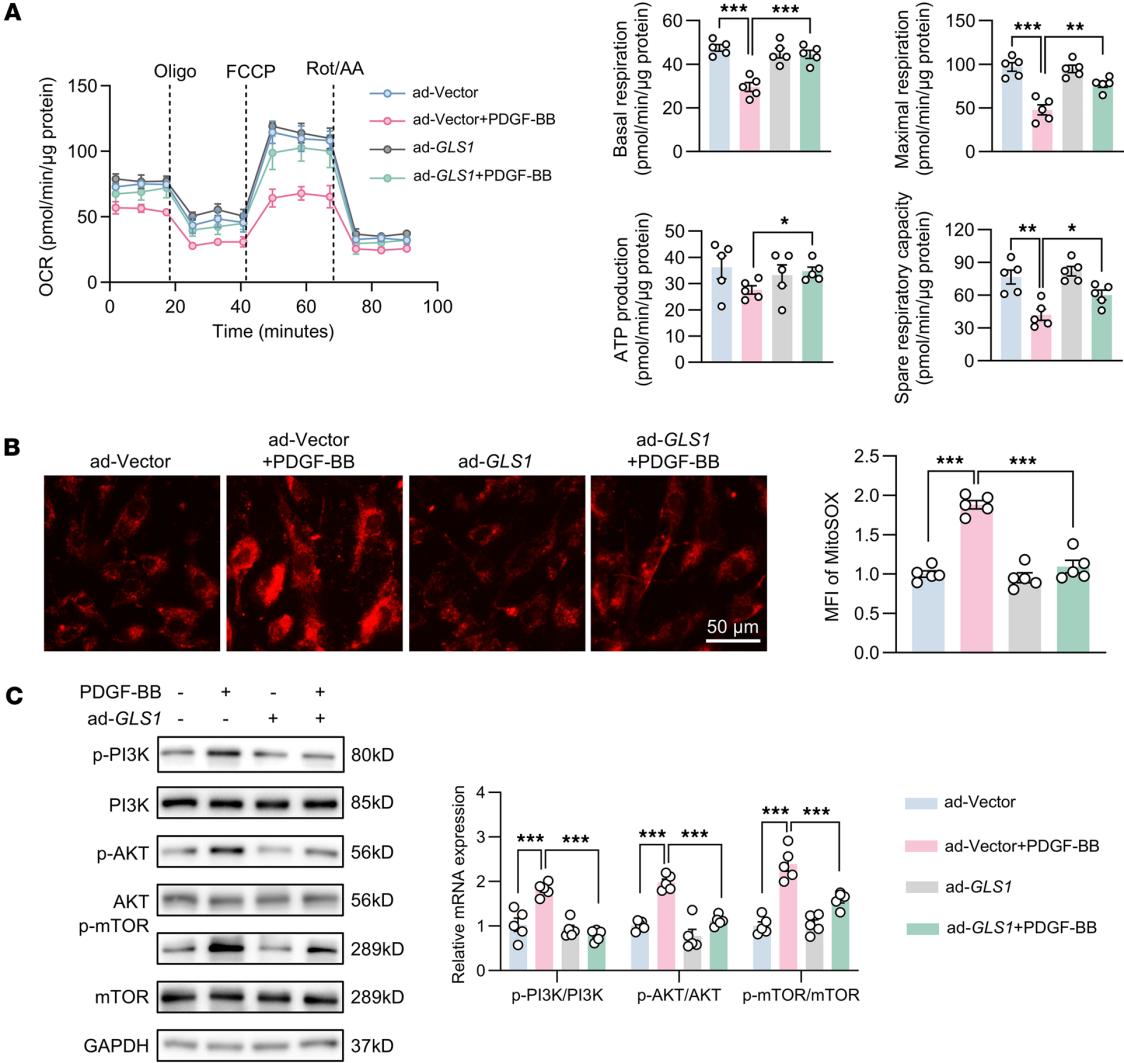
GLS1 modulates mitochondrial function and PI3K/AKT/mTOR signaling during VSMC phenotypic switching. (**A**–**C**) HASMCs were infected with adenovirus containing empty vector (ad-Vector) or GLS1-encoding plasmids (ad-*GLS1*), and then treated with PDGF-BB. (**A**) Summarized oxygen consumption rate (OCR) tracings in HASMCs. Basal OCR was measured, followed by sequential injections of 1 μM oligomycin, 1.5 μM FCCP, and a mixture of 2 μM rotenone and 2 μM antimycin A. (**B**) Mitochondrial reactive oxygen species were detected by MitoSOX staining in HASMCs. Scale bar: 50 μm. (**C**) Western blot analysis of phosphorylated and total PI3K, AKT, and mTOR levels in HASMCs. Data are presented as mean ± SEM. Statistical analysis was performed using 1-way ANOVA followed by Tukey’s multiple-comparison test (**B** and **C**). *P < 0.05, **P < 0.01, ***P < 0.001.

GLS1 plays an essential role by catalyzing the hydrolysis of glutamine to glutamate, a central metabolic intermediate with multifaceted functions (18). Glutamate serves as a precursor for α-ketoglutarate, which enters the TCA cycle to support mitochondrial ATP generation; and for glutathione synthesis, a critical antioxidant defense mechanism against oxidative stress (19, 20) (Figure 6C). Notably, mitochondrial dysfunction and disrupted glutathione homeostasis synergistically increase reactive oxygen species (ROS) accumulation. Elevated ROS levels can subsequently activate the PI3K/AKT signaling pathway (21). Therefore, we hypothesized that GLS1 may participate in the metabolism of glutamate to regulate PI3K/AKT signaling, and thereby modulate VSMC phenotypic switching. For verification, we assessed the cellular glutamate content and found that the glutamate level in HASMCs was reduced after PDGF-BB stimulation, which was increased by GLS1 overexpression (Figure 6D). Meanwhile, the levels of glutamate, glutathione, and α-ketoglutarate were suppressed upon PDGF-BB incubation, while overexpression of GLS1 restored the levels of glutathione and α-ketoglutarate to baseline values (Figure 6, E and F). Moreover, mitochondrial stress test showed that ad-*GLS1* transfection effectively improved PDGF-BB–impaired oxidative phosphorylation in VSMCs, protecting against mitochondrial dysfunction (Figure 7A). MitoSOX staining revealed that GLS1 overexpression mitigated the mitochondrial oxidative stress induced by PDGF-BB (Figure 7B). In parallel, the PDGF-BB–induced phosphorylation of PI3K, AKT, and mTOR was significantly attenuated by GLS1 overexpression (Figure 7C). These findings collectively demonstrated that GLS1 promotes the glutamate-derived glutathione and α-ketoglutarate contents to suppress ROS-related activation of the PI3K/AKT pathway, thereby improving VSMC phenotypic switching under pathological conditions.

### The expression of GLS1 is regulated by RARα.

**Figure 8:**
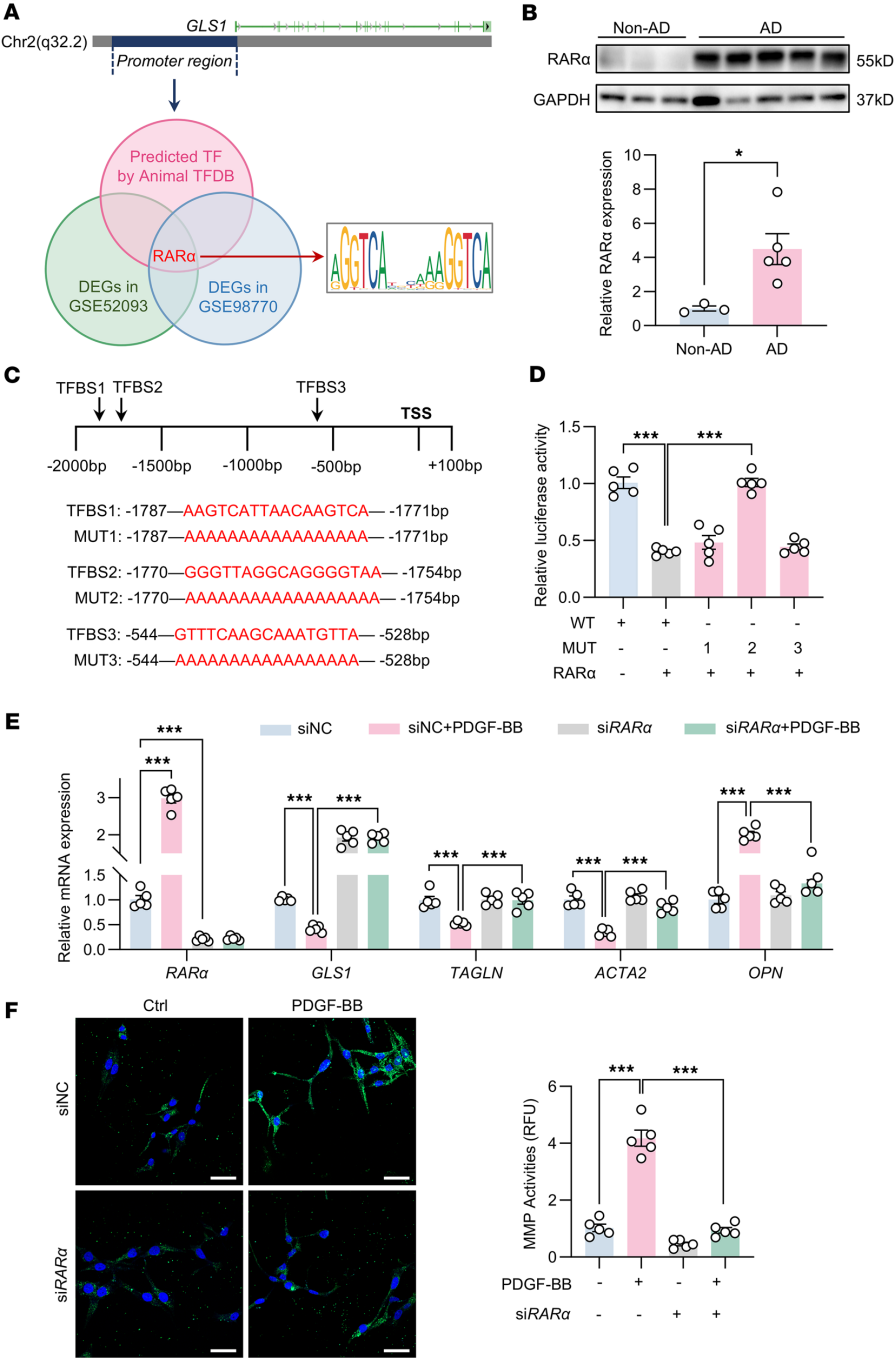
Transcription of GLS1 is regulated by the transcription factor RARα. (**A**) Prediction of *GLS1* promoter–binding transcription factors by AnimalTFDB and using the region from 2,000 bp upstream to 100 bp downstream of the GLS1 transcription start site (TSS) as the promoter region. Venn diagram of DEGs in human aortic dissection samples relative to normal controls from the NCBI GEO database (GSE52093 and GSE98770). (**B**) Western blot analysis of RARα expression in the aorta from non-AD groups and AD patients. (**C**) The construction of wild-type (WT) and 3 mutated luciferase reporter gene plasmids for *GLS1* promoter by mutation of 3 potential binding sites of RARα predicted by JASPAR. (**D**) Relative luciferase activity in HEK293 cells of luciferase reporter constructs containing *GLS1* promoter or its mutants transfected along with pRL-TK (internal control plasmid) followed by transfection with RARα-encoding plasmid. (**E** and **F**) HASMCs were transfected with siRNA against *RARα* (si*RARα*) or negative control (siNC), and then treated with PDGF-BB. (**E**) The mRNA expression of *RARα*, *GLS1*, *TAGLN*, *ACTA2*, and *OPN* in HASMCs was detected by real-time qPCR. (**F**) Immunofluorescence images of in situ zymography (DQ gelatin) in HASMCs. MMP activity (green) was quantified by immunofluorescence intensity. Scale bars: 50 μm. (Blue: DAPI.) Data are presented as mean ± SEM. Statistical analysis was performed using unpaired, 2-tailed Student’s *t* test (**B**), or 1-way ANOVA followed by Tukey’s multiple-comparison test (**D**–**F**). *P < 0.05, ***P < 0.001.

To further unravel the regulatory mechanism of GLS1 downregulation in the context of VSMC phenotypic switching, we integrated the prediction of *GLS1* promoter–binding transcription factors with the AnimalTFDB database and the DEGs from transcriptomic data of AD patients (GSE52093 and GSE98770). Retinoic acid receptor-α (RARα) was identified as a transcriptional regulator of *GLS1* (Figure 8A). We found that RARα expression was enhanced both in aortas from AD patients (Figure 8B) and in PDGF-BB–stimulated HASMCs (Supplemental Figure 5A). Next, we assessed the transcriptional effect of RARα on GLS1 by constructing luciferase reporter gene plasmids with mutation of the 3 potential binding sites of RARα from the JASPAR database (Figure 8C). The result of luciferase reporter gene assay revealed that mutation of transcription factor binding site 2 (TFBS2) effectively reversed the transcriptional inhibition of *GLS1* that resulted from RARα overexpression in both HEK293 cells and HASMCs (Figure 8D and Supplemental Figure 6A), suggesting that RARα should bind to the –1,770 to –1,754 bp of the *GLS1* promoter region to inhibit its transcription. Furthermore, the binding of RARα to the *GLS1* promoter was significantly enhanced upon PDGF-BB stimulation in HASMCs (Supplemental Figure 6B). Then, we used siRNA to knock down *RARα* to evaluate the effect of RARα on VSMC phenotypic switching (Supplemental Figure 7A). The quantitative PCR (qPCR) results showed that inhibition of *RARα* by siRNA could promote the expression of *GLS1* in HASMCs, while the decreased *ACTA2* and *TAGLN* and increased *OPN* mRNA levels were reversed by *RARα* knockdown in PDGF-BB–treated HASMCs (Figure 8E). Meanwhile, Western blot analysis yielded consistent results (Supplemental Figure 7B). Also, MMP activities induced by PDGF-BB were inhibited by RARα knockdown (Figure 8F). These data suggested that RARα inhibition could improve VSMC phenotypic switching through increasing GLS1 expression.

### The RARα inhibitor AR7 protects against BAPN-induced AD.

**Figure 9:**
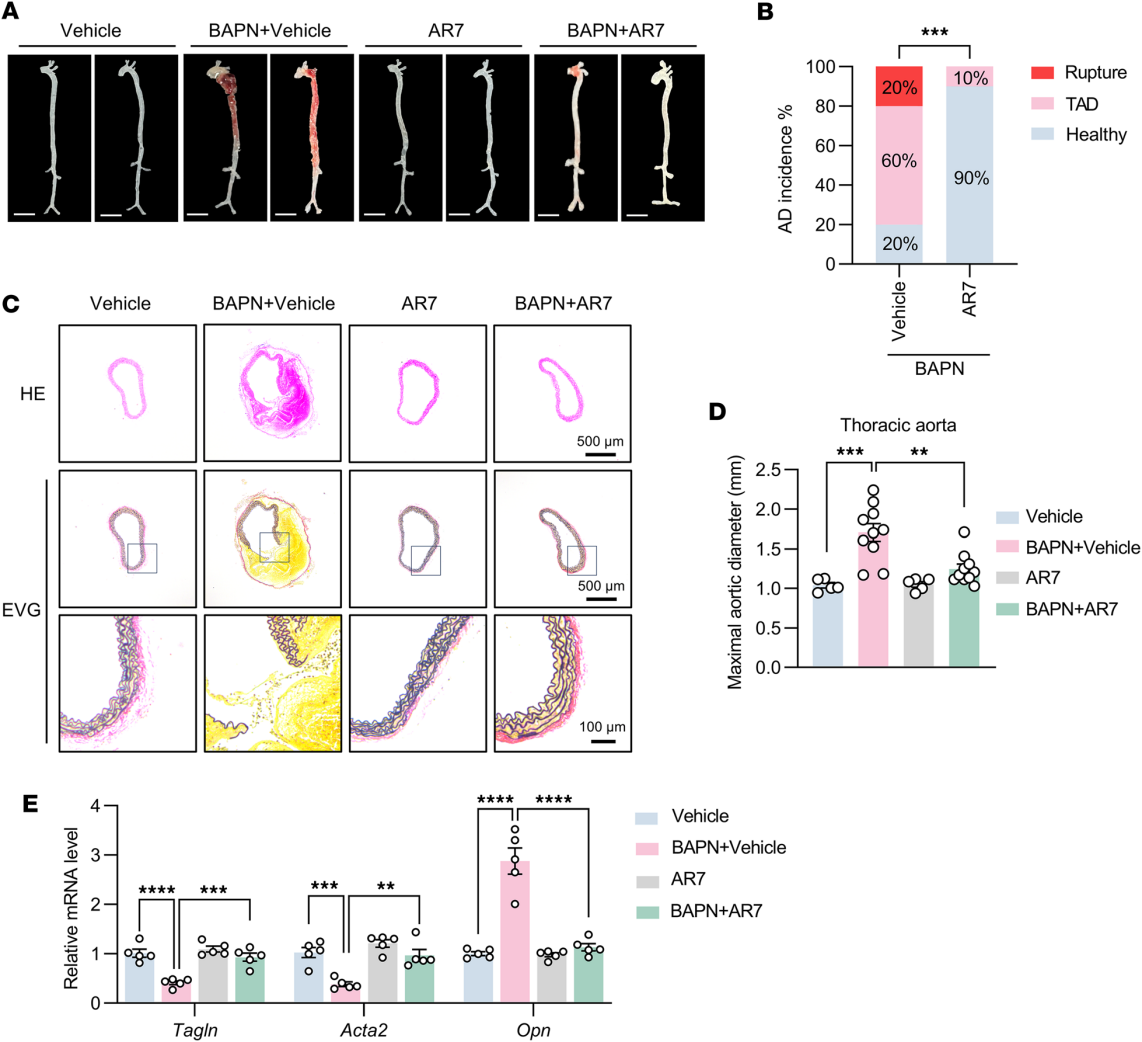
The RARα inhibitor AR7 attenuates AD in BAPN-treated mice. (**A**–**E**) Three-week-old male C57BL/6 mice were treated with BAPN in drinking water (1 g/kg/d) with or without intraperitoneal injection of AR7 (10 mg/kg/d) for 4 weeks. (**A**) Representative morphology of aortae from mice in the control group (*n* = 5) and the BAPN-treated group (*n* = 10). Scale bars: 5 mm. (**B**) The incidence of aortic dissection and rupture in BAPN-treated mice (*n* = 10). (**C**) H&E and EVG staining of aorta from control or BAPN-treated mice. Scale bars: 500 μm (top and middle panels), 100 μm (bottom panels). (**D**) Quantification of maximal diameters measured ex vivo of aorta from control and BAPN-treated mice. (**E**) The mRNA expression of *Tagln*, *Acta2*, and *Opn* in aorta from control and BAPN-treated mice was detected by real-time qPCR. Data are presented as mean ± SEM. Statistical analysis was performed using Fisher’s exact test (**B**), or 1-way ANOVA followed by Tukey’s multiple-comparison test (**D** and **E**). **P < 0.01, ***P < 0.001, ****P < 0.0001.

We next evaluated whether pharmacological targeting of the RARα/GLS1 axis could attenuate AD. Given the lack of available GLS1 agonists, we used AR7, a RARα inhibitor, to evaluate whether pharmacological blockade of RARα exhibits a protective role in AD. Three-week-old mice were fed with BAPN water and injected intraperitoneally with AR7 (10 mg/kg/d) for 4 weeks. We found that AR7 treatment mitigated the AD lesion (Figure 9A) and lowered the incidence of aortic dissection and rupture in mice fed with BAPN (Figure 9B). H&E and EVG staining showed that AR7 administration reduced aortic dilatation and improved media degeneration in the BAPN-induced mouse AD model (Figure 9C). AR7 also mitigated the maximal thoracic aorta diameter induced by BAPN (Figure 9D). The mRNA levels of VSMC phenotypic switching–related genes showed that AR7 treatment could reverse the reduced expression of *Tagln* and *Acta2* and enhanced expression of *Opn* in aorta from BAPN-fed mice (Figure 9E). Taken together, these data demonstrated that RARα inhibition via AR7 may represent a potential therapeutic strategy for AD.

## Discussion

This study establishes GLS1 as a crucial regulator in AD pathogenesis through comprehensive multiomics analyses, human tissue validation, and functional experiments. Our findings reveal that GLS1 maintains VSMC homeostasis by modulating glutamate metabolism. We further characterize the RARα/GLS1 transcriptional axis as a mechanistically important and therapeutically relevant pathway in AD. These results advance our knowledge of metabolic regulation in AD and identify potential targets for clinical intervention.

Glutamine metabolism exerts broad regulatory effects across diverse pathologies, including cancer and cardiovascular diseases (10, 22, 23). Under physiological conditions, extracellular glutamine undergoes cellular uptake and is converted by GLS1 into glutamate and ammonia. Glutamate serves dual metabolic functions: (a) it is transformed via transaminases or glutamate dehydrogenase into α-ketoglutarate, fueling the TCA cycle for ATP synthesis; and (b) it serves as a precursor for glutathione biosynthesis to maintain redox homeostasis (22). Our experimental data demonstrate that VSMC-specific *Gls1* overexpression significantly reduced dissection incidence in BAPN-treated murine models, attenuated medial degeneration, and produced concordant effects in cultured HASMCs. Notably, diminished intracellular glutamate levels during VSMC phenotypic switching align with clinical metabolomic profiling, which reveals markedly reduced plasma glutamate concentrations in thoracic AD patients compared with healthy controls (24). Existing evidence indicates that glutamine accumulation triggers synthetic phenotype transition in VSMCs (23), which aligns with our observation that metabolic dysregulation drives pathology. However, while Zhang et al. reported that the GLS1 inhibitor BPTES attenuated glutamine-induced dedifferentiation, we interpret this effect as a blockade of the specific microRNA-143/THY1 signaling pathway triggered by excess glutamine, rather than a reflection of metabolic benefit. In our pathological context, GLS1 downregulation leads to a depletion of glutamate and its derivatives (glutathione and α-ketoglutarate), creating a metabolic deficit that drives phenotypic switching. Thus, while excessive glutamine signaling may promote dedifferentiation via specific pathways like microRNA-143/THY1, sufficient GLS1 activity is required to maintain the metabolic homeostasis (glutamate/glutathione/α-ketoglutarate) necessary for the contractile phenotype. Consequently, GLS1 downregulation disrupts the glutamate/glutamine equilibrium, representing a distinct metabolic hallmark of AD pathogenesis. Nevertheless, prospective clinical studies should determine whether circulating glutamine/glutamate ratios could serve as predictive biomarkers for AD susceptibility.

The PI3K/AKT pathway orchestrates critical cellular processes including proliferation, differentiation, survival, and metabolism, with established roles in AD pathogenesis (25, 26) and VSMC phenotypic modulation (27). ROS are recognized mediators of aberrant PI3K/AKT activation (28). Our mechanistic studies establish that GLS1 attenuates pathological phenotype switching by restoring α-ketoglutarate and glutathione pools, thereby reducing ROS accumulation and preventing PI3K/AKT/mTOR hyperactivation. These findings position the GLS1-mediated metabolic signaling axis as a promising therapeutic target for AD intervention.

Existing cancer biology studies have identified transcriptional regulators of GLS1 such as cMyc (29) and cJun (30). In our work, an integrated bioinformatics analysis based on the AnimalTFDB database — which incorporates genomic data from 183 animal species and predicts transcription factor binding properties using optimized models and classification rules, achieving a manually curated prediction accuracy of 99.33% for human transcription factors (31) — highlighted retinoic acid receptor-α (RARα) as a putative upstream regulator of GLS1. RARα is a nuclear receptor–type transcription factor that plays key roles in embryonic development, metabolic regulation, cellular differentiation, and apoptosis (32), and it has been reported to exhibit oncogenic potential in multiple malignancies (33, 34). While retinoid signaling has been shown to be protective in vascular contexts, such as suppressing arterial calcification (35) and attenuating endothelial inflammation (36, 37), our findings uncover a distinct, context-specific role for RARα in AD. Here, we provide experimental evidence that RARα directly represses GLS1 transcription. Silencing RARα significantly restores GLS1 expression and attenuates VSMC phenotypic modulation. Critically, pharmacological inhibition of RARα with AR7 recapitulated these protective effects in vivo, reinstating the contractile phenotype of VSMCs in a BAPN-challenged mouse model. Collectively, these findings define RARα as a transcriptional suppressor that promotes VSMCs’ pathological remodeling in AD through downregulating GLS1, highlighting RARα blockade as a mechanistically grounded therapeutic strategy.

Several limitations in our study should be acknowledged. First, while the BAPN-induced murine model recapitulates salient histopathological features of AD, interspecies divergences persist in disease progression dynamics, inflammatory signatures, and metabolic profiles compared with human pathophysiology. Consequently, validation across human cohorts remains imperative before clinical translation. Second, only male mice were used in the in vivo experiments because of the known influence of estrogen on AD susceptibility. Therefore, the applicability of our findings to female mice remains to be determined. Future studies should address potential sex-specific differences in GLS1-mediated regulation of VSMC phenotypic switching and AD. Third, GLS1 regulation likely involves additional transcription factors beyond RARα as well as epigenetic modifications, which warrant further investigation. Further comprehensive analyses in diverse pathological contexts and expanded patient cohorts will be essential to rigorously evaluate GLS1’s therapeutic potential.

In summary, our findings establish that GLS1 maintains the contractile phenotype of VSMCs and mitigates AD through coordinated regulation of glutamate metabolism, attenuation of oxidative stress, and suppression of PI3K/AKT/mTOR signaling cascades. We further identify the RARα/GLS1 axis as a previously unrecognized therapeutic target and demonstrate that pharmacological blockade of RARα with AR7 exhibits translational promise for AD intervention. This work delineates a mechanistic basis for metabolic dysregulation in AD and provides a foundation for developing precision therapies targeting mitochondrial metabolism.

## Methods

### Sex as a biological variable.

Considering the influence of estrogen on AD, the mouse experiments were performed using male mice (38). Whether these findings are applicable to female mice remains unknown. For the human aortic tissue analysis, both male and female subjects were included in the AD and control groups (Supplemental Table 1).

### Human aortic tissue collection.

Human aortic tissues were collected with prior approval from the Medical Ethics Committee of Nanjing Drum Tower Hospital (Ethics ID 2020-046-01) and the Medical Ethics Committee of Zhongda Hospital (Ethics ID 2024ZDSYLL368-P01), in full compliance with the Declaration of Helsinki. Written informed consent was obtained from all participants. Ascending aortic samples were collected from patients with Stanford type A aortic dissection (AD; *n* = 8) during open surgical repair and from control patients without a history of AD undergoing coronary artery bypass graft surgery (non-AD; *n* = 5). Exclusion criteria included genetic aortic diseases, connective tissue disorders, infections, aortitis, and trauma-related disease. The characteristics of patients are given in Supplemental Table 1. After excision, periadventitial fat and thrombus were removed, and tissues were rinsed in saline, flash-frozen in liquid nitrogen, and stored at –80°C.

### Animal studies.

All procedures were approved by the Animal Ethics Committee (Approval 2407008) and followed International Council for Laboratory Animal Science guidelines.

*Tagln^Cre/+^* mice and *Gls1^loxP/loxP^* mice were obtained from the Model Animal Research Center of Nanjing University. A Cre-*loxP*–based targeting strategy was used to generate *Gls1*-conditional-knockout mice. To achieve gene deletion of *Gls1* specifically in VSMCs, *Tagln^Cre/+^* mice were crossed with *Gls1^loxP/loxP^* mice. The resulting *Gls1^loxP/loxP^* *Tagln^Cre/+^* mice were designated as smooth muscle cell–specific *Gls1*-knockout mice (*Gls1^SMKO^*), while *Gls1^loxP/loxP^* littermates (*Gls1^fl/fl^*) served as controls.

All mice were maintained under specific pathogen–free conditions (20°C–26°C, 30%–70% humidity, 12-hour light/12-hour dark cycle). Standard chow and water were provided ad libitum.

### Animal procedures.

For overexpression studies, the coding sequence of mouse *Gls1* (GenBank NM_001081081) was cloned in reverse orientation into the pLVX-FLEX-EF1α-ZsGreen lentiviral vector. This vector employs a double-floxed inverse orientation (FLEX) switch containing 2 pairs of heterotypic *loxP* sites. In the absence of Cre recombinase, the gene of interest remains silent. Upon Cre-mediated recombination in smooth muscle cells, the cassette undergoes inversion, placing the *Gls1* sequence in the correct orientation under the control of the EF1α promoter. A FLAG tag was fused to the *Gls1* coding sequence to facilitate detection. The integrity and reading frame of the final construct (pLVX-FLEX-FLAG-mGls) were verified by Sanger sequencing using the CMV-F primer (5′-CGCAAATGGGCGGTAGGCGTG-3′). For the control group, an empty pLVX-FLEX-EF1α-ZsGreen vector (Lenti-Vector) was used, which also expresses ZsGreen upon Cre recombination but lacks the *Gls1* insert. Constructs were packaged into lentiviral particles. Male 3-week-old *Tagln^Cre/+^* mice received tail vein injections of either Lenti-*Gls1* (5 × 10^9^ transducing unit in 200 μL) or Lenti-Vector, then were given β-aminopropionitrile (BAPN; Sigma-Aldrich, catalog A3134; 1 g/kg/d via drinking water) for 4 weeks to induce AD. Transduction efficiency was confirmed by detection of ZsGreen fluorescence in aortic sections, and exogenous GLS1 expression was verified using anti-FLAG antibodies.

For smooth muscle cell–specific *Gls1*-knockout mice, induction of AD at 3 weeks of age resulted in a relatively high rate of early mortality. Therefore, 5-week-old mice were used for subsequent experiments and were given BAPN (Sigma-Aldrich, catalog A3134; 0.5 g/kg/d via drinking water) to induce AD (39).

For pharmacological intervention, male *Tagln^Cre/+^* mice received BAPN as described above and intraperitoneal administration of AR7 (MedChemExpress, catalog HY-101106; 10 mg/kg in saline; stock in DMSO, Beyotime, catalog ST038) for 4 weeks. The dosage of AR7 was selected based on published literature (40).

Mice were anesthetized with 5% isoflurane until the loss of pedal reflex was confirmed, then euthanized by CO_2_ inhalation, in compliance with the American Veterinary Medical Association Guidelines for the Euthanasia of Animals.

### Transcriptomic and single-cell data analysis.

Microarray datasets GSE52093 (5 controls, 7 AD) and GSE98770 (5 controls, 6 AD) were downloaded from the Gene Expression Omnibus (GEO). Data were processed in R using the limma package with robust multi-array average for background correction, quantile normalization, and probe annotation. Differentially expressed genes (DEGs) were defined as *P* < 0.05 and |log_2_FC| > 1.

Single-cell RNA sequencing data (GSE213740) were processed with the Seurat package. Cells with 200 < nFeature_RNA < 2,500 and mitochondrial content less than 5% were retained. Principal component analysis and JackStraw (*P* < 0.001) guided dimensionality reduction. Clustering used the Louvain algorithm (resolution = 0.5), and visualization was via UMAP. The characteristics of GEO datasets are described in Supplemental Table 2.

### Transcription factor binding site prediction.

The *GLS1* promoter region (–2,000 bp to +100 bp relative to transcription start site) was retrieved from NCBI (GRCh38). Potential transcription factor binding sites were identified via the AnimalTFDB database (31) and intersected with DEGs from GSE52093 and GSE98770. Candidate factor binding sites were confirmed using the JASPAR database (41).

### Cell culture and treatments.

Human aortic smooth muscle cells (HASMCs; ScienCell, catalog 6110) were cultured in Smooth Muscle Cell Medium (ScienCell, catalog 1101) supplemented with 2% fetal bovine serum (FBS), smooth muscle growth supplement, and antibiotics (100 U/mL penicillin/streptomycin) at 37°C, 5% CO_2_, and used up to passage 8. HEK293 cells (ATCC, catalog CRL-3216) were grown in DMEM with 10% FBS (Gibco, catalog 10099).

For stimulation, HASMCs were serum-starved in DMEM without FBS for 12 hours, followed by stimulation with PDGF-BB (MedChemExpress, catalog HY-P7055) at the indicated concentration (20 ng/mL) for 24 hours. siRNAs for GLS1 and RARα (GenePharma) were transfected using Lipofectamine 3000 (Invitrogen, catalog L3000075), with scrambled siRNA as negative control.

### Western blotting.

Proteins from tissues or cells were extracted in RIPA lysis buffer (Beyotime, catalog P0013B) containing protease inhibitors (Thermo Fisher Scientific, catalog 78438). Protein concentrations were determined using the BCA assay (Thermo Fisher Scientific, catalog 23225). Equal amounts were separated by SDS-PAGE, transferred to PVDF membranes (Millipore, catalog ISEQ00010), blocked with 5% nonfat milk for 2 hours, and incubated with primary antibodies (Supplemental Table 3) overnight at 4°C, followed by HRP-conjugated secondary antibodies (Supplemental Table 3) for 2 hours at room temperature. Detection was via ECL reagent (Thermo Fisher Scientific, catalog 34094) and quantification with ImageJ (NIH).

### Quantitative real-time PCR.

Total RNA was isolated using TRIzol reagent (Takara, catalog 9109), reverse-transcribed using HiScript II Q RT SuperMix (Vazyme, catalog R222-01), and amplified on a QuantStudio system (Thermo Fisher Scientific) using SYBR Green Master Mix (Vazyme, catalog Q131-02). *GAPDH* served as reference; relative expression was calculated using the 2^–ΔΔCt^ method. Primer sequences are in Supplemental Table 4.

### Chromatin immunoprecipitation assay.

Chromatin immunoprecipitation (ChIP) assays were performed using the ChIP Assay Kit (Beyotime, catalog P2078). Formaldehyde-cross-linked chromatin was sonicated and incubated with anti-RARα antibody overnight at 4°C. Immune complexes were captured with protein A/G agarose, washed, eluted, and reverse-cross-linked at 65°C. DNA was purified for qPCR analysis.

### Immunofluorescence staining of paraffin-embedded human aortic tissue.

Sections were deparaffinized with xylene and rehydrated in a graded ethanol series (100%, 95%, 90%, 80%, 75%) and double-distilled water. Antigen retrieval was performed by heating in citrate buffer (Beyotime, catalog P0081), followed by permeabilization with 0.3% Triton X-100 (Beyotime, catalog ST795) for 10 minutes and blocking with 10% BSA (Beyotime, catalog ST023) for 1 hour. Primary antibodies against GLS1 and ACTA2 (Supplemental Table 3) were applied overnight at 4°C, followed by Alexa Fluor 488– or 594–conjugated secondary antibodies (Supplemental Table 3) for 1 hour at room temperature. Nuclei were counterstained with DAPI (SouthernBiotech, catalog 0100-20). Images were captured on a confocal microscope (Zeiss LSM 800).

Mouse aortae were embedded in OCT compound (Sakura, catalog 4583), cryosectioned at 8 μm (Leica, catalog CM1950), fixed with 4% paraformaldehyde (Servicebio, catalog G1101) for 20 minutes, permeabilized with 0.3% Triton X-100, blocked with 10% BSA, and stained as above. Paraffin-embedded tissues were sectioned at 5 μm and stained with H&E (Beyotime, catalog C0105S) or Verhoeff–van Gieson (Abcam, catalog ab150667) according to manufacturer protocols. Optical images were obtained using an Olympus microscope (Olympus, catalog U-HGLGPS).

### MMP activity assay.

In situ gelatinase activity was assessed using the EnzChek Gelatinase/Collagenase Assay Kit (Thermo Fisher Scientific, catalog E12055). HASMCs were treated according to the manufacturer’s instructions and incubated at 37°C for 24 hours. The fluorescence of DQ gelatin is quenched until MMP-catalyzed hydrolysis occurs. The resulting fluorescence intensity is directly proportional to proteolytic digestion. Cells were fixed with 4% paraformaldehyde and stained with DAPI. Proteolytic activity was detected as green fluorescence (at 495 nm absorption/515 nm emission) by confocal microscopy (Zeiss LSM 800). Five fields of view were randomly selected per experiment. Quantification was performed according to the average green fluorescence intensity (active MMPs) by ImageJ, and the values were normalized relative to the control group.

### Dual-luciferase reporter assay.

Wild-type and mutant *GLS1* promoters were synthesized by Tsingke Biotechnology and cloned into the pGL6 luciferase reporter vector (Beyotime, catalog D2091). Recombinant pGL6 dual-luciferase reporter plasmids were cotransfected with or without RARα-encoding plasmid (YouBio, catalog G111296) into HEK293T cells. Luciferase activity was measured using the Dual-Luciferase Reporter Assay System (Promega, catalog E1910) according to the manufacturer’s instructions.

### Mitochondrial stress test.

Oxygen consumption rate (OCR) was measured using a Seahorse XF96 Analyzer (Agilent Technologies, Santa Clara, CA, USA). HASMCs were seeded in 1% gelatin–coated Seahorse 96-well plates (Agilent Technologies, Santa Clara, CA, USA) at a density of approximately 20,000 cells per well. Cells were cultured for 24 hours, followed by adenovirus transfection. The next day, cells were stimulated with PDGF-BB for 24 hours. OCR was measured in DMEM (containing 10 mM glucose, 2 mM glutamine, and 1 mM pyruvate, pH 7.4). Sequential additions of 1.0 μM oligomycin, 1.5 μM FCCP, and a mixture of 2 μM rotenone and 2 μM antimycin A were made to assess the metabolic profile. Basal respiration, maximal respiration, ATP production, and spare respiratory capacity were calculated as previously described (42).

### Mitochondrial superoxide detection.

Cells were incubated with MitoSOX Red (Beyotime, catalog S0061S) for 60 minutes at 37°C, washed twice with PBS (Beyotime, catalog C0221A), and imaged under a fluorescence microscope or Zeiss LSM 800 confocal system. The mean fluorescence intensity (MFI) of MitoSOX Red per cell was quantified using ImageJ software and normalized to the MFI of the control groups.

### Measurement of glutamate, α-ketoglutarate, and glutathione.

Glutamate, α-ketoglutarate, and glutathione were measured using a Glutamate Assay Kit (Solarbio, catalog BC1580), an α-Ketoglutarate Assay Kit (Beyotime, catalog S0323S), and a Glutathione Assay Kit (Beyotime, catalog S0053), respectively.

### Statistics.

Data are presented as mean ± SEM. Statistical significance was set at *P* < 0.05. Normality was tested using the Shapiro-Wilk test, and non-parametric tests were used for non-normal distributions. Two-group comparisons were made using 2-tailed Student’s *t* test or Welch’s *t* test. For multiple-group comparisons, variance homogeneity was first tested using Brown-Forsythe, and 1-way ANOVA or Welch’s ANOVA was applied. All in vitro experiments were performed with at least 3 independent biological replicates, and each experiment included triplicate technical replicates. GraphPad Prism 9 was used for statistical analysis and graphing.

### Study approval.

The use of human tissues was approved by the Medical Ethics Committees of Nanjing Drum Tower Hospital (Ethics ID 2020-046-01) and Zhongda Hospital (Ethics ID 2024ZDSYLL368-P01), and written informed consent was obtained from all participants. Animal experiments were conducted in accordance with the Animal Research: Reporting of In Vivo Experiments (ARRIVE) guidelines and approved by the Animal Ethics Committee of Nanjing Medical University (approval 2407008).

### Data availability.

The RNA sequencing data were deposited in the NCBI’s GEO database (GSE324926). Publicly available datasets analyzed in this study include GSE52093, GSE98770, and GSE213740. Underlying data summarized for all figures can be found in the Supporting Data Values XLS file. Additional data are available upon request.

## Author contributions

HC, TS, and SZ conceived and designed the study and revised the manuscript. WX and CN analyzed the data and wrote the initial draft. WX and CN performed the experiments. CL reanalyzed the RNA sequencing data. HC, TS, SZ, and DW supervised the experiments. The order of co–first authors is based on the amount of work contributed.

## Conflict of interest

The authors have declared that no conflict of interest exists.

## Funding support

- National Natural Science Foundation of China (grants 82370262, 82500498, 82570476).
- Natural Science Foundation of Jiangsu province (BK20250767).

## Supplementary Material

Supplemental data

Unedited blot and gel images

Supporting data values

## Version 1 04/23/2026

In-Press Preview

## Version 2 06/08/2026

Electronic publication
