# Supplementary material for: GLS1 governs vascular smooth muscle cell phenotypic switching and aortic dissection via glutamate metabolism
Source: JCI Insight. 2026 Apr 23;11(11):e203575. doi: 10.1172/jci.insight.203575 (PMC13313552; doi:10.1172/jci.insight.203575)
Supplement: Supplemental data [file jciinsight-11-203575-s071.pdf]

# Supplemental Material

## GLS1 governs vascular smooth muscle cell phenotypic switching and aortic dissection via glutamate metabolism

**Authors**

Wei Xie[1,#]; Chen Ning[3,#]; Chen Lu[1]; Dongjin Wang[4]; Shuang Zhao[3,*]; Tianyu Song[2,*]; Hailong Cao[1,*].

**Affiliations**

[1]Department of Cardiac Surgery, Zhongda Hospital, School of Medicine, Southeast University, Nanjing, Jiangsu, China;

[2]School of Medicine, Nanjing University of Chinese Medicine, Nanjing, Jiangsu, China;

[3]School of Pharmacy, Nanjing Medical University, Nanjing, Jiangsu, China.;

[4] Department of Cardiac Surgery, Nanjing Drum Tower Hospital, Affiliated Hospital of Medical School, Nanjing University, Nanjing, Jiangsu, China.

* Corresponding author

[#] These authors contributed equally

***Corresponding author:**

*Hailong Cao, MD, PhD.

Department of Cardiac Surgery, Zhongda Hospital, School of Medicine, Southeast University.

Address: No. 87 Dingjiaqiao, Gulou District, Nanjing, Jiangsu Province, 210009, China

Tel: +86-25-83262261

E-mail: hailongcao_zd@163.com; hailongcao@seu.edu.cn

*Tianyu Song, PhD.

School of Medicine, Nanjing University of Chinese Medicine.

Address: No. 138 Xianlin Avenue, Qixia District, Nanjing, Jiangsu Province, 210023, China

Tel: +86-15250966190
E-mail: tianyusong@njucm.edu.cn;

*Shuang Zhao, PhD.
School of Pharmacy, Nanjing Medical University.
Address: No. 101 Longmian Ave, Jiangning District, Nanjing, Jiangsu, 211166, China
Tel: +86-15720800551
E-mail: zhaoshuang@njmu.edu.cn.

## Supplemental Tables

## Supplemental table S1. The characteristics of patients

| Patients | Age (years) | Gender | Smoking | BMI (kg/m$^2$) | Hypertension | Diabetes mellitus | Hyperlipidemia |
|---|---|---|---|---|---|---|---|
| Non-AD1 | 58 | Male | No | 23.1 | Yes | No | No |
| Non-AD2 | 67 | Female | Yes | 26.7 | No | No | Yes |
| Non-AD3 | 54 | Female | No | 22.9 | Yes | Yes | Yes |
| Non-AD4 | 62 | Male | Yes | 25.3 | No | No | No |
| Non-AD5 | 63 | Female | No | 24.5 | No | No | No |
| AD1 | 59 | Male | No | 24.8 | Yes | No | Yes |
| AD2 | 68 | Female | Yes | 27.2 | No | No | No |
| AD3 | 55 | Male | No | 23.6 | Yes | No | No |
| AD4 | 72 | Female | Yes | 25.9 | Yes | Yes | No |
| AD5 | 60 | Female | No | 26.0 | Yes | No | Yes |
| AD6 | 64 | Male | No | 24.2 | Yes | No | No |
| AD7 | 57 | Female | Yes | 25.5 | No | No | No |
| AD8 | 63 | Male | No | 23.8 | Yes | No | No |

| Variables | Non-AD (n=5) | AD(n=8) | *P* value |
|---|---|---|---|
| Age (years, Mean ± SD) | 60.8 ± 5.0 | 62.3 ± 5.7 | 0.65 |
| Male (N, %) | 2 (40%) | 4 (50%) | 1.00 |
| Smoking (N, %) | 2 (40%) | 3 (37.5%) | 1.00 |
| BMI (kg/m$^2$, Mean ± SD) | 24.5 ± 1.6 | 25.1 ± 1.2 | 0.44 |
| Hypertension (N, %) | 2 (40%) | 6 (75%) | 0.29 |
| Diabetes mellitus (N, %) | 1 20%) | 1 (12.5%) | 1.00 |
| Hyperlipidemia (N, %) | 2 (40%) | 2 (25%) | 1.00 |

**Supplemental table S2. The characteristics of GEO datasets**

| GEO accession | Platform | Organism | Samples |
|---|---|---|---|
| GSE52093 | GPL10558 | Homo sapiens | 5 normal human aortas and 7 aortas from AD patients |
| GSE98770 | GPL14550 | Homo sapiens | 5 transplant donor aortas and 6 aortas from AD patients |
| GSE213740 | GPL18573 | Homo sapiens | 3 normal human aortas and 6 aortas from AD patients |

**Supplemental table S3. Primary and secondary antibodies used in this study**

| Target | Host pecies | Supplier | Catalog No. |
|---|---|---|---|
| GLS1 | Rabbit polyclonal | Proteintech | 12855-1-AP |
| RARα | Rabbit monoclonal | Abcam | ab275745 |
| TAGLN | Mouse monoclonal | Abcam | ab14106 |
| ACTA2 | Rabbit monoclonal | Abcam | ab124964 |
| OPN | Rabbit polyclonal | Abcam | ab8848 |
| ACTA2 | Mouse monoclonal | Santa Cruz Biotechnolog | sc-53015 |
| GAPDH | Mouse monoclonal | Abways Technology | AB0037 |
| β-Actin | Mouse monoclonal | Abways Technology | AB0035 |
| FLAG tag | Mouse monoclonal | Proteintech | 66008-3-Ig |
| PI3K | Rabbit monoclonal | Cell Signaling Technology | 4257T |
| AKT | Rabbit monoclonal | Cell Signaling Technology | 4691T |
| mTOR | Rabbit monoclonal | Cell Signaling Technology | 2983T |
| Phospho-AKT (Ser473) | Rabbit monoclonal | Cell Signaling Technology | 4060T |
| Phospho-mTOR (Ser2448) | Rabbit monoclonal | Cell Signaling Technology | 5536T |
| Phospho-PI3K | Rabbit monoclonal | Affinity Biosciences | AF-3241 |
| Alexa Fluor® 594 anti-mouse IgG | Donkey polyclonal | Thermo Fisher Scientific | A21203 |
| Alexa Fluor® 594 anti-rabbit IgG | Donkey polyclonal | Thermo Fisher Scientific | A21207 |
| Alexa Fluor® 488 anti-mouse IgG | Donkey polyclonal | Thermo Fisher Scientific | A21202 |
| Alexa Fluor® 488 anti-rabbit IgG | Donkey polyclonal | Thermo Fisher Scientific | A21206 |
| HRP-conjugated anti-mouse IgG | Goat polyclonal | Thermo Fisher Scientific | 31430 |
| HRP-conjugated anti-rabbit IgG | Goat polyclonal | Thermo Fisher Scientific | 31460 |

**Supplemental table S4. The primer sequences used for qRT-PCR analysis**

| Gene | Forward (5' to 3' sequence) | Reverse (5' to 3' sequence) | Species |
|---|---|---|---|
| *GLS1* | TCTACAGGATTGCGAACGTCT | CTTTGTCTAGCATGACACCATCT | Human |
| *RARα* | GGGCAAATACACTACGAACAACA | CTCCACAGTCTTAATGATGCACT | Human |
| *GAPDH* | GGAGCGAGATCCCTCCAAAAT | GGCTGTTGTCATACTTCTCATGG | Human |
| *ACTA2* | AAAGCAAGTCCTCCAGCGTT | TAGTCCCGGGGATAGGCAAA | Human |
| *TAGLN* | GGAAACCCACCCTCTCAGTC | TGCACTAGCCAAGTCATCCG | Human |
| *OPN* | ATCTCCTAGCCCCACAGACC | CACACTATCACCTCGGCCAT | Human |
| *Gls1* | GATGGCCCTAGGATGTCTGC | GCTGACTTGCCCACTCTCAT | Mouse |
| *Rarα* | TTCTTTCCCCCTATGCTGGGT | GGGAGGGCTGGGTACTATCTC | Mouse |
| *18S* | AGTCCCTGCCCTTTGTACACA | CGATCCGAGGGCCTCACTA | Mouse |
| *Opn* | AATCTCCTTGCGCCACAGAA | GGACATCGACTGTAGGGACG | Mouse |
| *Acta2* | CGCCTCCAGTTCCTTTCCAA | AGAGGGGGCCACCCTATAAT | Mouse |
| *Tagln* | AGGGGTGACATCACTGCCTA | GACTGCACTTCTCGGCTCAT | Mouse |

## Supplemental Figures and Figure Legends

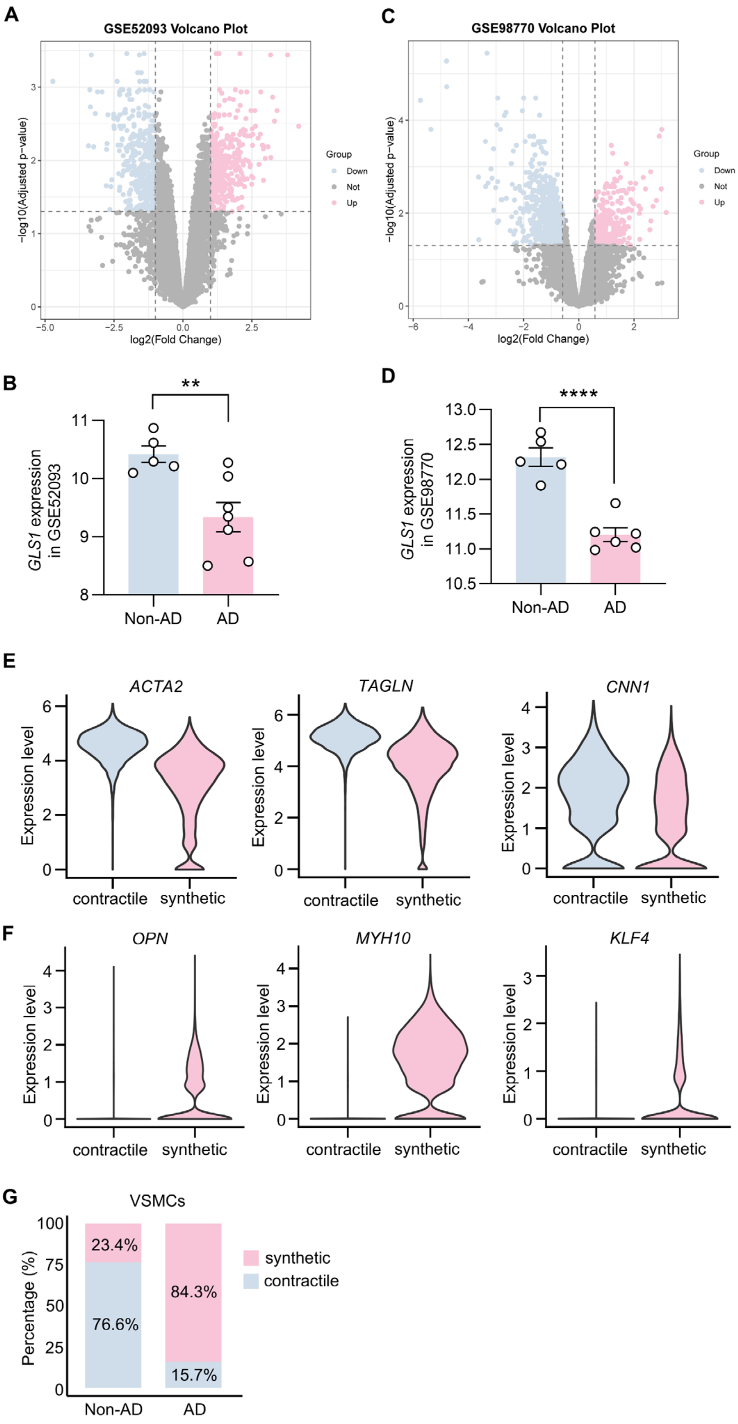


**Supplemental Figure S1. GLS1 expression is reduced in aortic dissection.**
**(A)** Volcano plot illustrating differentially expressed genes in human aortic dissection database (GSE52093), with upregulated (red) and downregulated (blue) genes indicated. **(B)** The relative *GLS1* expression in the aorta of Non-AD and AD patients according to GSE52093. **(C)** Volcano plot illustrating differentially expressed genes in human aortic

dissection database (GSE98770), with upregulated (red) and downregulated (blue) genes indicated. **(D)** The relative *GLS1* expression in the aorta of Non-AD and AD patients according to GSE98770. **(E)** The expression of contractile markers (ACTA2, TAGLN, CNN1) in synthetic VSMCs (cluster_2) and contractile VSMCs (cluster_5). **(F)** The expression of synthetic markers (OPN, MYH10, KLF4) in synthetic VSMCs (cluster_2) and contractile VSMCs (cluster_5). **(G)** The proportion of contractile and synthetic VSMCs in Non-AD and AD groups. Data are presented as mean ± SEM. Statistical analysis was performed using unpaired, two-tailed Student's *t* test (**C** and **D**). **$p < 0.01$, ****$p < 0.0001$.

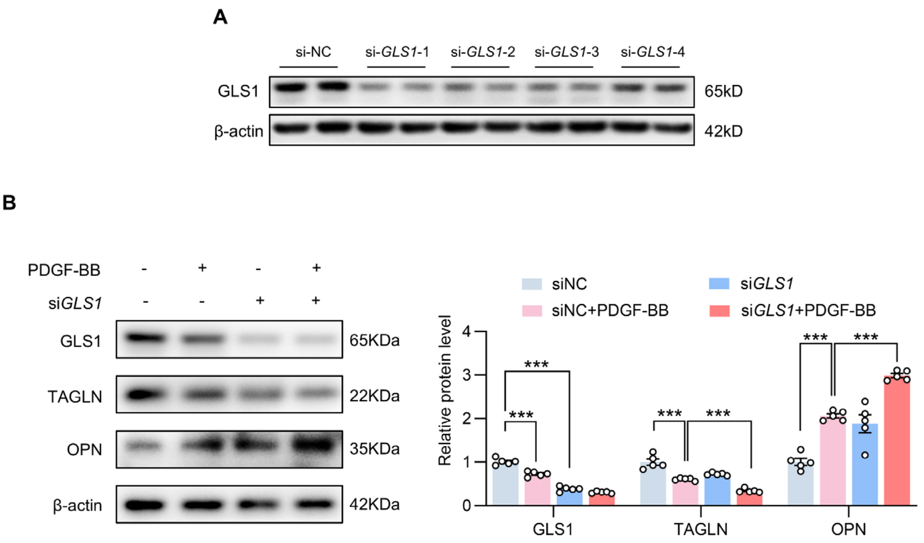


**Supplemental Figure S2. *GLS1* knockdown aggravates VSMCs phenotypic switching.**

**(A)** The efficiency of si*GLS1* in HASMCs was detected by Western blot. **(B)** HASMCs were transfected with siRNA against *GLS1* (si*GLS1*) or negative control (siNC), and then treated with PDGF-BB. Western blot and quantitative analysis of GLS1, TAGLN and OPN expression in HASMCs. Data are presented as mean ± SEM. Statistical analysis was performed using one-way ANOVA followed by Tukey's multiple-comparison test (**B**). ***$p < 0.001$.

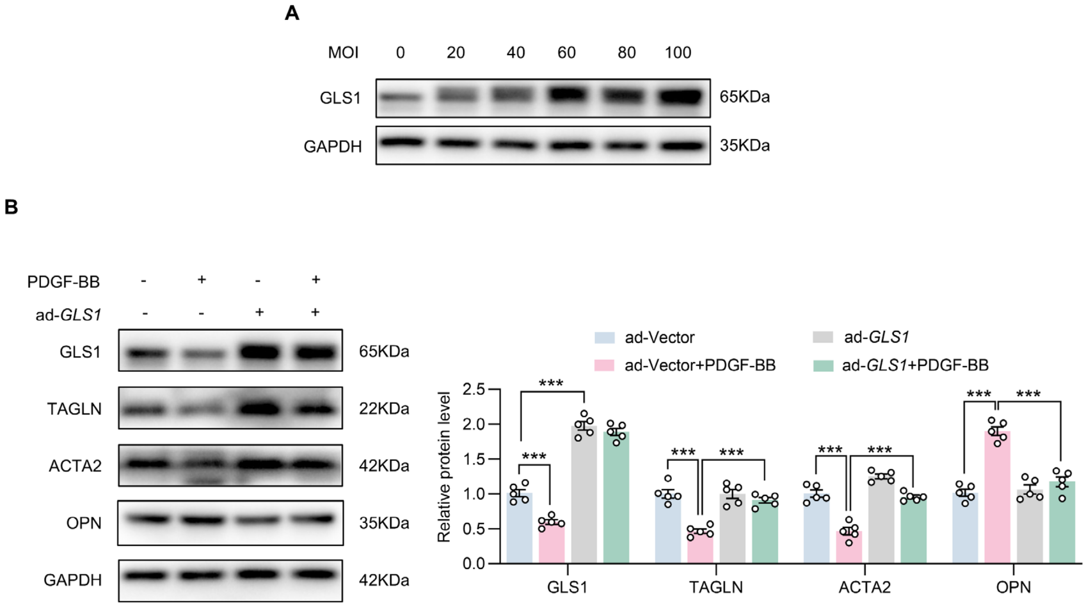


**Supplemental Figure S3. GLS1 overexpression alleviates VSMCs phenotypic switching.**

**(A)** The efficiency of different concentration of adenovirus mediated GLS1 overexpression in HASMCs was detected by Western blot. **(B)** HASMCs were infected with adenovirus containing empty vector (ad-Vector) or GLS1-encoding plasmids (ad-*GLS1*), and then treated with PDGF-BB. Western blot and quantitative analysis of GLS1, TAGLN, ACTA2 and OPN expression in HASMCs. Data are presented as mean ± SEM. Statistical analysis was performed using one-way ANOVA followed by Tukey's multiple-comparison test (**B**). ***$p < 0.001$.

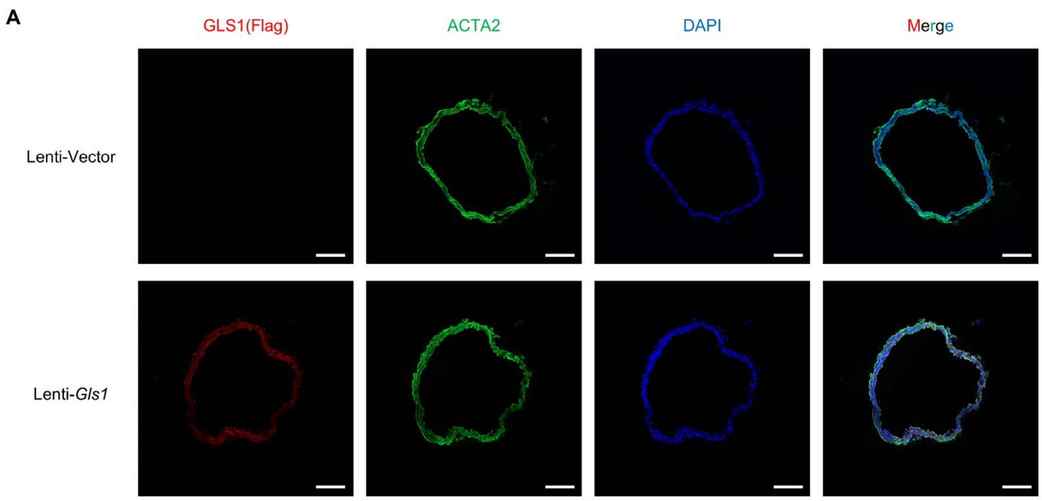


**Supplemental Figure S4. The efficiency of exogenous GLS1 expression in VSMCs of aorta.**

**(A)** Three-week-old male *Tagln*$^{Cre/+}$ mice were intravenously injected with lentivirus containing control vector or reverse *Gls1* sequence with two *loxP* sites. The expression of exogenous GLS1 was detected by immunofluorescence staining for Flag (red), ACTA2 (green) and DAPI (blue) in mice aorta sections. Scale bars, 200 μm. n = 3.

A

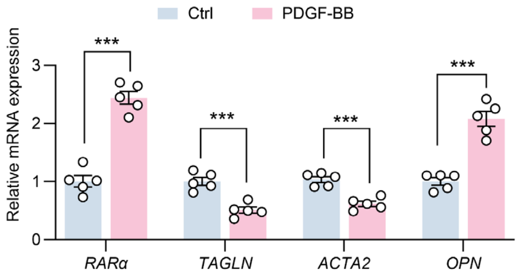


**Supplemental Figure S5. RARα expression is increased in PDGF-BB treated HASMCs.**

**(A)** HASMCs were treated with PDGF-BB. The mRNA expression of *RARα*, *TAGLN*, *ACTA2* and *OPN* in HASMCs was detected by qRT-PCR. Data are presented as mean ± SEM. Statistical analysis was performed using unpaired, two-tailed Student's *t* test (**A**). ***p < 0.001.

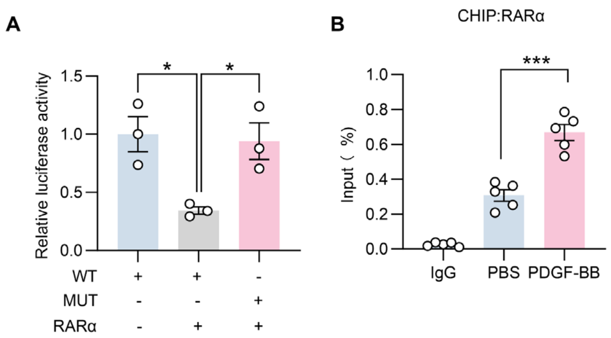


**Supplemental Figure S6. Verification of the binding of RAR to *GLS1* promoter.** **(A)** Relative luciferase activity in HASMCs co-infected with adenoviruses carrying either the wild-type *GLS1* promoter or its transcription factor binding site 2 (TFBS2) mutant luciferase reporter constructs, along with adenovirus expressing RARα. **(B)** HASMCs were treated with PDGF-BB. Chromatin immunoprecipitation (ChIP) assays were performed with IgG or anti- RARα antibody, followed by qRT-PCR with primers targeting *GLS1* promoter regions. Data are presented as mean ± SEM. Statistical analysis was performed using one-way ANOVA followed by Tukey's multiple-comparison test (**A**) and unpaired, 2-tailed Student's *t* test (**B**). $^{*}p < 0.05$, $^{***}p < 0.001$.

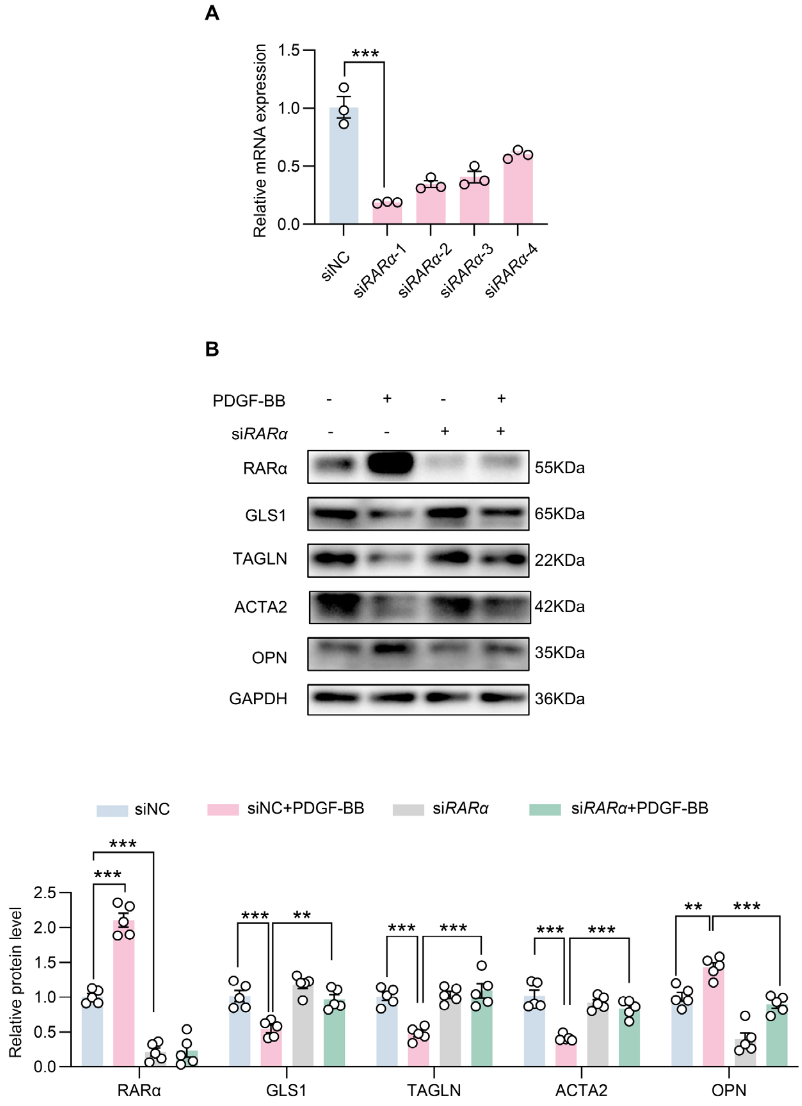


**Supplemental Figure S7. *RARα* knockdown alleviates VSMCs phenotypic switching.**

**(A)** The efficiency of si*RARα* in HASMCs was detected by qRT-PCR. **(B)** HASMCs were transfected with siRNA against *RARα* (si*RARα*) or negative control (siNC), and then treated with PDGF-BB. Western blot analysis of RARα, GLS1, TAGLN, ACTA2 and OPN expression in HASMCs. Data are presented as mean ± SEM. Statistical analysis was performed using one-way ANOVA followed by Tukey's multiple-comparison test (**C**). **p < 0.01, ***p < 0.001.
