# Supplementary material for: GLS1 governs vascular smooth muscle cell phenotypic switching and aortic dissection via glutamate metabolism
Source: JCI Insight. 2026 Apr 23;11(11):e203575. doi: 10.1172/jci.insight.203575 (PMC13313552; doi:10.1172/jci.insight.203575)

Figure 1B

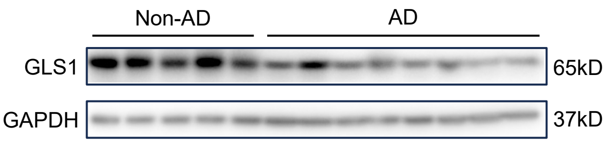

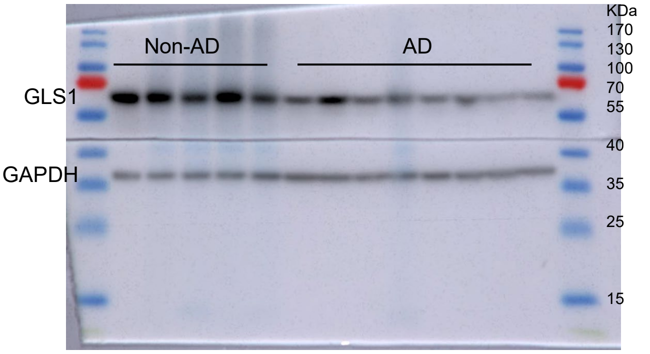

Figure 2A

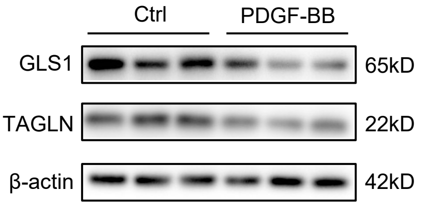

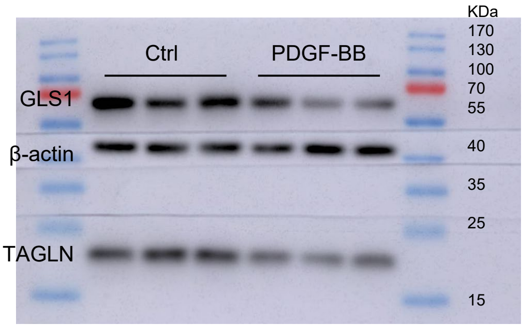

Figure 7I

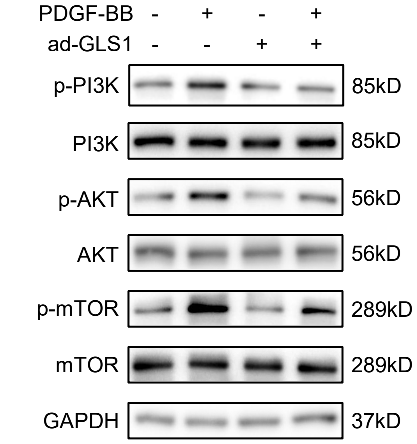

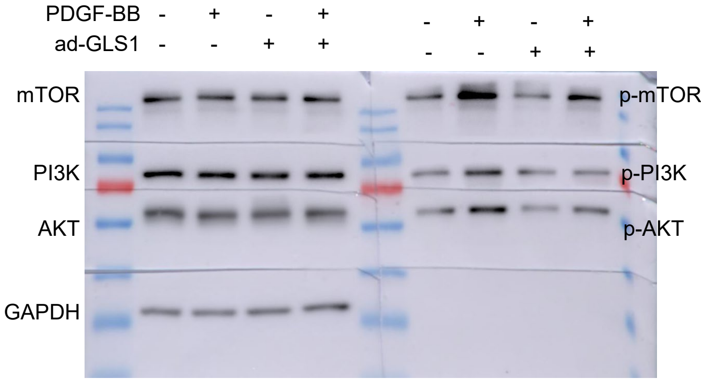

Figure 8B

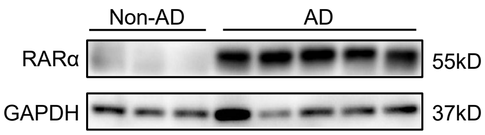

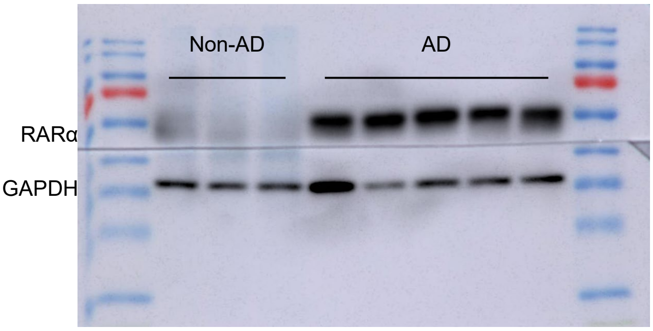

Supplement Fig 2A

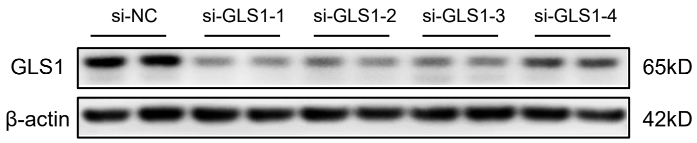

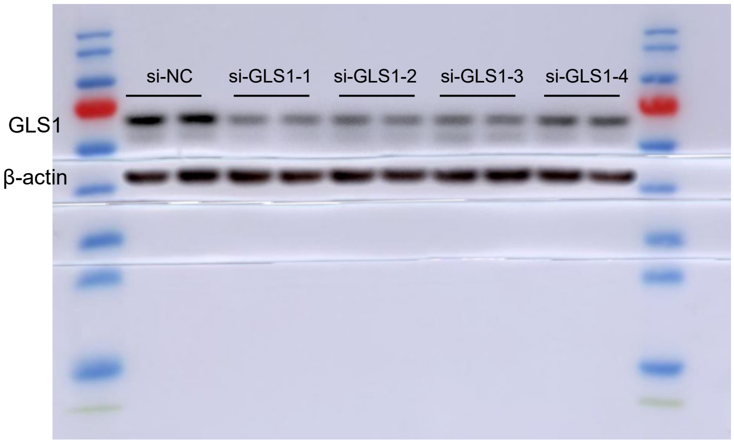

Supplement Fig 2B

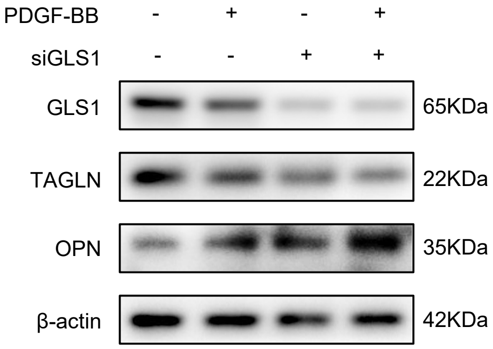

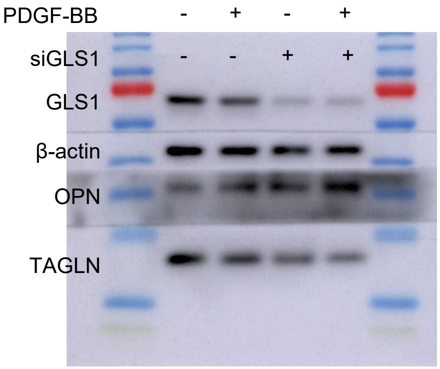

Supplement Fig 3A

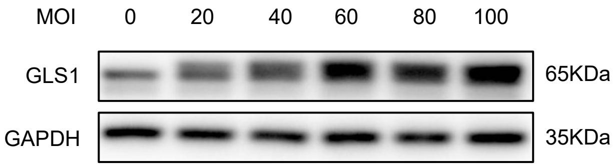

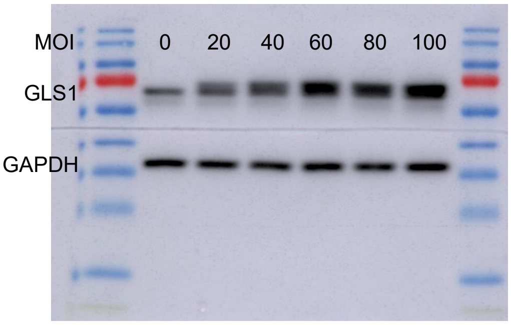

Supplement Fig 3B

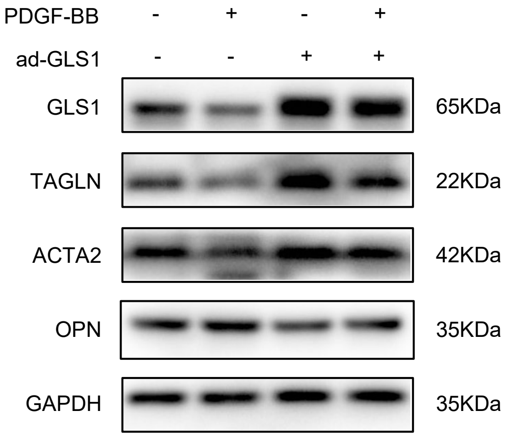

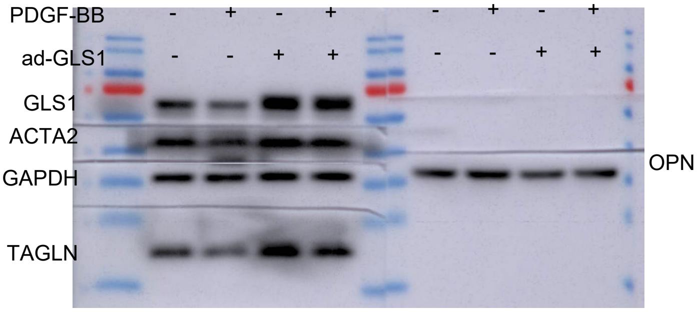

Supplement Fig 7B

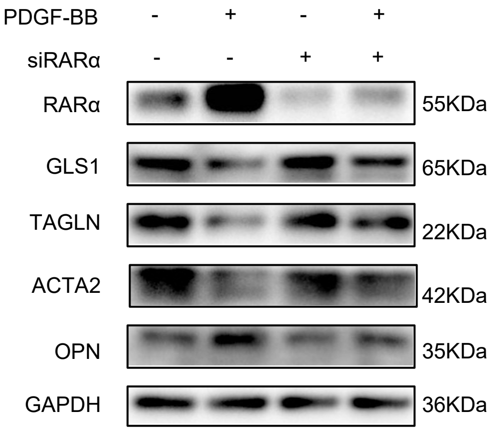

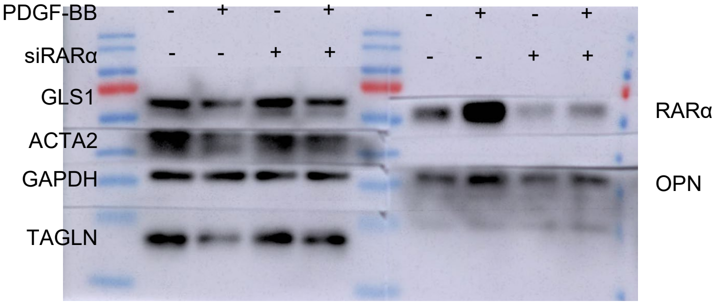

Supplement: Unedited blot and gel images [file jciinsight-11-203575-s072.pdf]
